# Becoming a neurosurgeon in France: A qualitative study from the trainees’ perspective

**DOI:** 10.1016/j.bas.2023.102674

**Published:** 2023-09-29

**Authors:** Bertrand Debono, Clément Baumgarten, Antoine Guillain, Nicolas Lonjon, Olivier Hamel, Anne-Hélène Moncany, Elsa Magro

**Affiliations:** a Department of Neurosurgery, Paris-Versailles Spine Center, Hôpital Privé de Versailles, Les Franciscaines, 78000, Versailles, France; b Department of Neurosurgery, University Hospital of Grenoble, Grenoble, France; c AMADES (medical Anthropology, Development and Health), Centre de la Vieille Charité, 2 rue de la Charité, Marseille, France; d Department of Neurosurgery, Hôpital Gui de Chauliac, Montpellier University Medical Center, Montpellier, France; e Department of Psychiatry and Addictive Behaviour, Gerard Marchant Hospital Center, Toulouse, France; f Department of Neurosurgery, Ramsay-Clinique des Cèdres, Cornebarrieu, France; g Department of Neurosurgery, CHU Cavale Blanche, INSERM UMR 1101 LaTIM, Brest, France

**Keywords:** Mentoring, Neurosurgical training, Qualitative research, Operating rooms, Surgical competencies, Resident, Trainee, Trainee surgeon

## Abstract

**Introduction:**

The training of neurosurgeons is evolving in a world of socio-professional changes, including the technological revolution, administrative pressure on stakeholders, reduced working hours, geographical heterogeneity, generational changes, to name but a few.

**Research question:**

This qualitative study aimed to explore experiences and feedback of French neurosurgical trainees concerning their training.

**Material and methods:**

The grounded theory approach was used with 23 neurosurgical trainees’ interviews. Inclusion was continued until data saturation. Six researchers (an anthropologist, a psychiatrist, and four neurosurgeons) thematically and independently analyzed data collected through anonymized interviews.

**Results:**

Data analysis identified three superordinate themes: (1) The Trainee-Senior Dyad, where the respondents describe a similar bipolarity between trainees and faculty (trainees oscillating between those who fit into the system and those who are more reluctant to accept hierarchy, faculty using an ideal pedagogy while others refuse to help or invest in training); (2) The difficulty to learn (describing pressure exercised on trainees that can alter their motivation and degrade their training, including the impact of administrative tasks); (3) A pedagogy of empowerment (trainee’ feelings about the pertinent pedagogy in the OR, ideal sequence to progress, progressive empowerment especially during the shifts, and stress of envisioning themselves as a senior neurosurgeon).

**Discussion and conclusion:**

Respondents emphasize the heterogeneity of their training both intra- and inter-university-hospital. Their critical analysis, as well as the formalization of their stress to become autonomous seniors, can be an important link with the reforms and optimizations currently being carried out to improve and standardize the training of young French neurosurgeons.

## Highlights

• Qualitative studies offer valuable approach to exploring trainees' feedback on their education.
• They emphasize the heterogeneity of their theorical and practical education process.
• The primary interaction with senior surgeons is at the center of their preoccupation.
• Trainees claim to be able to cope with tough working conditions if there are qualitative returns.
• A critical analysis of their feedbacks can be a crucial link with the reforms and optimizations in their training.

## 1 Introduction

The training of neurosurgeons is evolving in a world of constant social and professional mutations, and despite advancements in the surgical education paradigm, it is crucial to determine whether future physicians are being educated to be independent and self-confident surgeons (Lipsman et al., 2017; Fontes et al., 2014; Bernstein et al., 2006).

Technological revolution, administrative pressure on different stakeholders, reduced time available for education, generational changes in aspirations and perspectives—all these elements impact the training of future neurosurgeons, evolving on a continuum between students and practitioners (Louie et al., 2019; Majmundar et al., 2021; Chikwe et al., 2004).

The bibliography on surgical pedagogy, mentoring, and empowerment in surgery is extensive (Gkiousias, 2021; Johannink et al., 2016; Lin and Reddy, 2019; Möller et al., 2008). Based on Halstead's fundamental model, many studies depict attempts to unify neurosurgical curriculum and possible customizations to the personality and learning abilities of each individual (Lipsman et al., 2017; Kerr and O'Leary, 1999). The neurosurgical trainee integrates prolonged and complex training, which will develop beyond the operating room, in a world that oscillates between enabling and hindering variables and in a very demanding setting in terms of stress, workload, and rising responsibility (Zaed et al., 2020; Yaeger et al., 2020).

While it is obvious that a trainee's training should prepare him/her to become a neurosurgeon in the holistic sense of the term, this challenging objective necessitates close collaboration with faculty, integration into a team, and adherence to an institution with stringent regulations (Akhigbe et al., 2017; Jensen et al., 2018; Knifed et al., 2008).

Despite the significant commitment of the French College of Neurosurgery, neurosurgical training in France remains defined by disparities between university training centers, based on inter-regional academic faculties (Campus de Neurochirurgie). This heterogeneity remains a major challenge for the national university-hospital system, one of whose primary goals is to unify teaching and training high-level neurosurgeons within a few years.

Qualitative studies detailing trainees' experiences of their training are still rare; however, their interaction with their seniors, peers, and the institutions in which they work, as per their own experience can provide a useful body of testimony for assessing the current situation. These studies highlight the positive elements or factors that necessitates optimization, and integrate their feedback into current improvement efforts (Knifed et al., 2008; Neal et al., 2022; Bello et al., 2018; Stienen et al., 2016a, 2016b, 2016c). Therefore, this qualitative study explored the experiences of French neurosurgical trainees and their comments on their educational system.

## 2 Materials and methods

### 2.1 Participants and sampling

Since our project aimed to investigate the perceptions of neurosurgical trainees about the different dimensions of their medical learning, we selected grounded theory as the overall framework for the study (Starks and Brown Trinidad, 2007). The use of semi-structured interviews allows respondents to have less limited thematic development angles than with serial questioning.

**Fig. 1:**
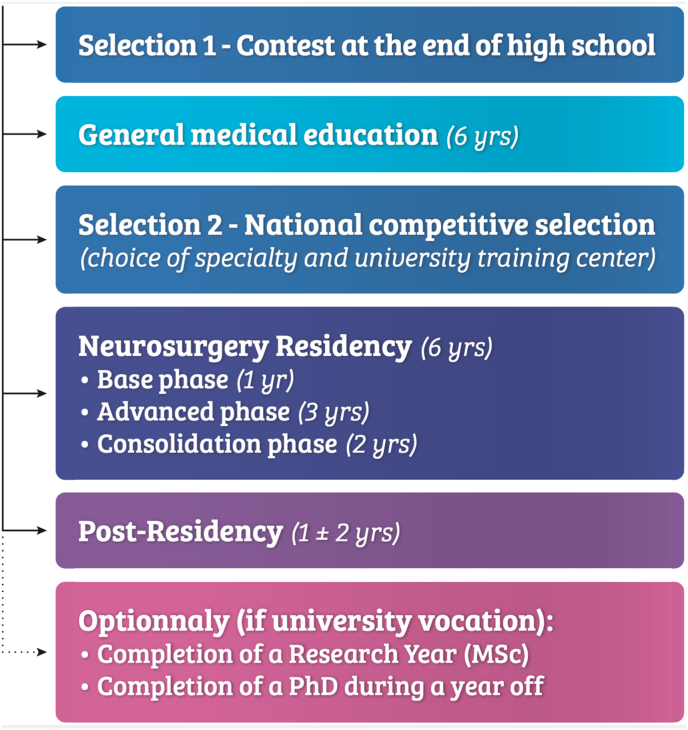
Training curriculum for a French neurosurgery trainee (2023).

Fig. 1 provides an overview of the medical curriculum of French neurosurgery trainees.

Interviews were performed between November 2021 and April 2022. A theoretical purposive sampling technique using maximum variation was adopted following the grounded theory methodology (Guillain et al., 2020). We favored trainees who had completed at least six semesters of neurosurgery specialization (out of a total of 10) to acquire more insightful feedbacks on their training, but we selected trainees of different ages, genders, experiences, and geographical locations of training. We initially compiled a list of 30 trainees, but saturation of themes occurred after 23 interviews (i.e., no new themes appeared in the analysis of the interviews), thereby defining the definite sample (Morse, 1995).

### 2.2 Data collection and analysis

The unstructured interviews were conducted by a researcher skilled in qualitative research who was unfamiliar with the respondents. The mean length of interview was 48.25 min (range 31.48–76.03 min).

An independent secretary anonymized the interviews before transcribing them. Each verbatim was read repeatedly, allowing for a thematic analysis in several stages. The first step was coding verbatim excerpts, trying to stay close to the respondents' words, to isolate elementary units of meaning. These initial codings were then grouped into categories according to their similarities. Furthermore, they were grouped into sub-themes and superordinate themes. We thus followed the inductive principles of the Grounded Theory to build the thematic from the observations and not from pre-established hypotheses. The themes were defined independently by researchers of different backgrounds (an anthropologist, a psychiatrist, and four surgeons) so that we could compare various perspectives with our own backgrounds and pay systematic attention to our own effects as researchers at each stage of the process (Malterud, 2001). Consensus was established in meetings attended by two other surgeons to finalize the triangulation. The analysis process was performed using NVIVO software (QSR International, Melbourne, Australia).

The Ethics Committee of the French College of Neurosurgery and the Data Protection Authority (CNIL, *Commission Nationale de l'Informatique et des Libertés*) approved the study (IRB number: IRB00011687), and the trainees interviewed provided their written consent. Our research protocol follows the Consolidated Criteria for Reporting Qualitative Research (COREQ) statement (Tong et al., 2007).

Prior to the interviews, the respondents provided their written informed consent after being told that all data would be anonymized and that records would not be archived at the end of the study.

## 3 Results

### 3.1 Participants data

Theme saturation occurred after the 23rd interview (14 men and 9 women). All participants were from university centers representative of all the regions of our country and had completed at least six semesters of training out of a possible ten.

### 3.2 Thematic areas

**Fig. 2:**
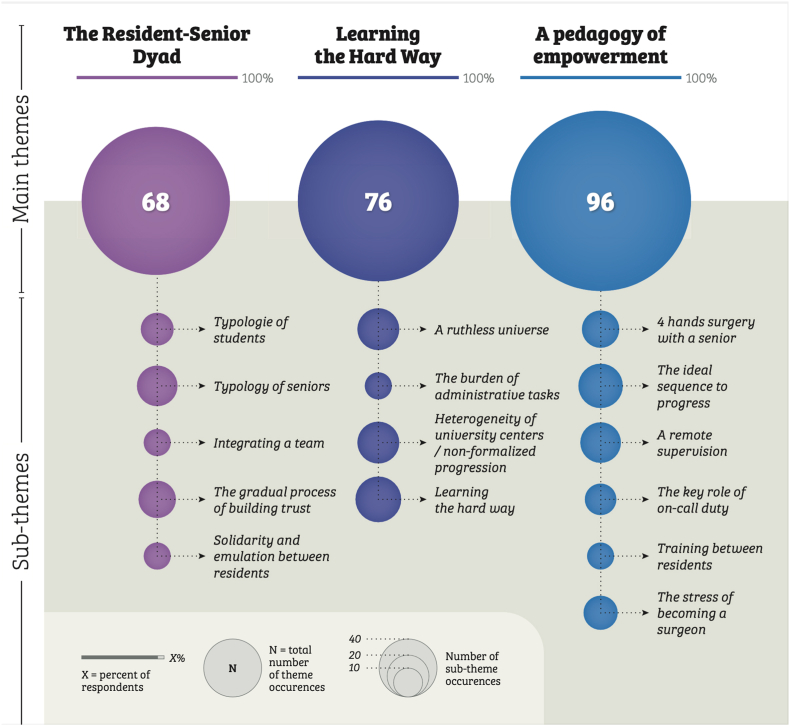
Description of the main themes and subthemes.

Data analysis revealed three superordinate themes based on recurring elements in interviews (Fig. 2). The themes are analyzed in detail in the following section, and selected quotes from the interviews are reported to support our findings, with more examples in S1 File (Online Resource). Quotations are translations that closely capture the essence of the spoken language.

### 3.3 Trainee-senior dyad

#### 3.3.1 Typology of students

Respondents describe the hierarchical nature of their university, where it is necessary to fit in and take initiative to avoid being regarded as inattentive or incompetent.

*The trainees who struggle are the ones who … well … who don’t seem to realize that the university hospital operates within an extremely hierarchical system that you have to adapt to in order to fit in (#11_3)*

Many trainees understand that fostering the ideal learning environment begins with the student's own attitude.

*Some trainees create the right conditions for their residency- they are eager to learn, they check the operations schedule for the next day, show an interest in what’s going on, read the patient reports, do their research. Then there’s another type of trainees … the ones who just rock up on the day and breeze into the operating room like “hi, um … I’m here to help”- it usually doesn’t work out so well for them because the seniors don’t like that (#13_1)*

In fact, several respondents raise concerns about a new generation of trainees who claim their entitlement before proving themselves.

*We’re seeing a new generation of trainees nowadays who are slightly different … perhaps … well, they act more entitled, but the thing is … they feel entitled before they’ve even proven themselves! (#9_4)*

#### 3.3.2 Typology of seniors

Most respondents emphasize the heterogeneity in the teaching quality of the seniors with whom they interact.

*Well, for me there’s no such thing as a good or bad way of teaching, but … at least when it comes to the surgeons we work with, well, we know they don’t all do things the same way. [#9_12]*

They describe a typology that ranges from the “non-helpers” to those who will guide the trainee step by step through the surgical learning process.

*There are seniors who, even after years of experience, even at their age, even though they’ve done the same operation a thousand times, still won’t let you do anything [#19_14]*

*I feel like … it’s really difficult for many of the senior surgeons … some will let you have a go, they’ll show you first, but then if you can’t do it, they take back the instruments. Then there are some who do everything themselves, they explain what they’re doing, but won’t let you have a go … so you think you’ve understood, but when the day comes to perform the operation and you’re standing there with the tools in your hand, well … you realize you can’t actually do it … Ultimately, there are very few senior surgeons out there who are able to describe everything they’re doing whilst also allowing you to practice with the instruments … that’s like the Holy Grail [#14_13]*

All respondents put the senior's patience as the most essential quality needed for teaching in the OR, as well as the willingness to educate and awareness of their own prior training.

*For the mentoring system to work in practice, surgeons need to be patient, but, well … you can’t change human nature … (#11_23)*

*I think the best trainers I had were people who were patient and, more importantly, people who hadn’t lost sight of how they got to where they are now … (#13_24)*

This results in a selection made by the trainees during the OR schedules, who sort out and choose to assist the surgeons who play the game while leaving the surgeons who do not help, who then find themselves operating without trainees.

*Senior surgeons who didn’t want to … teach me or let me have a go … well, they didn’t get to see what I could do. I focused on finding those who were … well, more keen to transmit their knowledge. (#19_36)*

#### 3.3.3 Integrating a team

Integration into a team and a hospital structure in the broadest sense is highlighted as an essential step in order to follow a quality curriculum and interact with the various hospital stakeholders, most notably senior surgeons involved in the training of trainees.

*Surgery isn’t just about what goes on in the operation room, that’s a big part of it -probably the most important part- but … it’s also about managing pre and post operation patient care, family relationships, and also interactions with the nurses, supervisor, nursing assistants, staff … (#11_6)*

Respondents describe a strategy to create their position and then develop their personality by proving that they have the potential to become a safe and reliable practitioner.

*First you have to prove that you’re not dangerous, then show that you’re useful … and then you have to prove you’ve got what it takes to become a surgeon. (#10_8)*

#### 3.3.4 Gradual process of building trust

Access to learning necessitates the development of a relationship of trust with the instructor, a gradual process that might take a long period of time.

*Mentoring obviously implies a certain level of … trust and complicity between mentor and mentee, so … of course we won’t all have the same experience of the mentoring system … it depends on your personality. [#3_20]*

*It's a gradual learning curve … until you’re ready to actually take over in the operating room. It takes hours and hours and countless operations together. I also think our trainers see our progress on a daily basis, helping them until eventually, it’s their turn to help us- in any case, in this hospital, you have to prove your worth [#10_19]*

Interaction with several seniors within the same team leads to relationships that are variably fruitful according to elective affinities.

*I have my mentor … and I think every trainee has a senior who they feel closer to than others … which isn’t necessarily a bad thing- there are always going to be different affinities [#10_18]*

#### 3.3.5 Solidarity and emulation between trainees

This universe, which may initially be hostile and individualistic, can benefit from mutual support between trainees.

*We’re all in the same boat so … well … if someone is struggling, we’re there to help, offer support, explain things. [#1_10]*

Mutual emulation is also the result of interaction between trainees of the same team.

*Yes, I think there’s also bit of emulation, when you see a peer from the year above performing an interesting procedure on their own, well … you can’t wait to be able to do the same. [#19_9]*

### 3.4 Learning the hard way

*When it comes to the training process … well, I think it’s important to highlight that … you’re not often allowed to say no. [#10_83]*

#### 3.4.1 Ruthless universe

The respondents emphasize a daily strain on life that can be traced back to a surgical tradition of demanding oneself and others, imposing a wide range of working hours and a harmful working environment with a heavy hierarchy. This aspect may be a legacy of an old conception of the surgical commitment.

*And there is a sort of … unwritten rule in surgery … that you have to be there all the time, never get tired, never complain, etc, make your presence known, never take breaks, …at least that’s always been the case for the older generation, but things need to change, and they are starting to change, which is a good thing. (#12_84)*

Some university centers are known to be unhelpful to young colleagues in training.

*Because if you have nothing to do … uh … if you’re just standing around in the hospital with nothing to do, then there’s not really much point … I knew which places to avoid, cities with a bad reputation because they overwork the trainees, don’t respect all the working conditions, take advantage … those guys are there from 7am to 9pm every day … (#23_86)*

This system is also home to senior staff with rough personalities that can degrade interpersonal relationships.

*Sometimes you have to just take things in your stride and accept that they’re a pain in the backside … and try not to take anything too personally … but they’re not all like that. (#6_85)*

This educational pressure would model a personality type that combines seriousness and self-sacrifice to suit the profile of an “ideal trainee” within a demanding hospital-university setting.

*And also to demonstrate that we have … a good management in the ward, of the workload, of the perioperative, of the preoperative, that we are … how to say … that we are dedicated, that we have an abnegation … well at work … that we are serious and stable, that's it. (#E15_66)*

#### 3.4.2 Burden of administrative tasks

Most trainees report a heavy administrative burden, which is a shift of tasks within the hospital structure.

*In (City A), trainees are increasingly treated like … secretaries (#14_89)*

This situation can be an obstacle to the quality of their professional training.

*The way hospitals are run can actually undermine medical practice and trainee training … because of, well … the huge administrative burden and myriad tasks to be carried out … which kind of compromise the smooth running of the teaching process (#5_90)*

#### 3.4.3 Heterogeneity of university centers/non-formalized progression

*It’s not well organised, you know, the mentoring system in the operating room is a bit … well, it’s a bit ad hoc, I would say. The senior surgeons only let the trainee perform a procedure if and when it suits them- there’s no organised system in place and that’s a shame- I think that’s the biggest weakness in the French system. (#16_95)*

Respondents identify a heterogeneity within the training centers, some of which are extremely formative and others where the trainee operates infrequently and late in his curriculum.

*I always thought that during a residency you would be able to operate from time to time, I didn’t realise that it depends on the hospital … in (City B) for example there’s not really the same … teaching philosophy … so, uh … I was a bit surprised to find out that … well … I wouldn’t be operating at all at the beginning of my residency: I was only allowed to watch. (#20_93)*

Most respondents report a geographic heterogeneity in surgical training, with some departments being very permissive and others relegating the trainee to an observation role.

*you either end up in a service like mine which is very permissive, where you are able to do things on your own very quickly, or … in another service, where you can’t do anything or touch anything until the end of your fifth, sixth, seventh semester- there don’t seem to be any … standard rules (#13_43)*

These geographic differences suffer from a lack of national guidance on individual progression through predefined stages, and students may dispute the academic mission of senior staff in teaching departments.

*It would be good to see more involvement in the training process, because a lot of the practitioner-professors … well, they never teach classes, they don’t offer any support, they don’t really do anything, so … well, it makes you wonder why they are even professors in the first place … (#16_44)*

The investment of the trainee in his or her own training may therefore be affected.

*I’m convinced that in any case the level of training isn’t homogenous- there are cities where you get better training, and cities where the training is less good. Of course, it also depends on the trainee: teaching is only half of the equation, but you have to determine which factors are conducive to quality teaching or not … (#11_94_94)*

#### 3.4.4 Learning the hard way

*And that’s how you learn … because unfortunately learning is a painful … and difficult process (#1_99)*

These impeding factors may result in an unfavorable feeling with qualitatively impactful working and learning environments.

*I knew that it was a very challenging field of work, but, well … the quality of life isn’t great, and I guess I wasn’t expecting … well, to have to make quite so many sacrifices. (#15_100)*

The trainees emphasize that they do not begrudge the amount of work inherent in their training; however, they regret a daily routine where quality degradation can alter their motivation.

*I would much rather work seventy hours and do three on-call shifts with cool senior surgeons who I get on well with than work forty hours, bored out of my mind, getting yelled at, only doing paperwork, visits, hardly any operations … basically all the boring stuff (#23_97)*

*I started to lose motivation at the hospital … it turned out to be a temporary phase, but that’s when I realised that the issue wasn’t the quantity but rather the quality of the work I was doing (#7_98)*

### 3.5 Pedagogy of empowerment

*In the operation room you have to abide by certain codes … everything is like a sort of choreography … it’s almost always the same thing … and when you first arrive you don’t know all of the … steps. So it’s complicated to begin with but gradually you start to understand it better and get into the swing of things, until eventually … well, you start doing things yourself … you start setting the tempo … so you basically go from being a total observer to … well, someone who is really able to take initiatives (#21_67)*

#### 3.5.1 Four hands surgery with a senior

Most trainees emphasize the importance of interacting with a senior surgeon as the basis of surgical training.

*I think that … well, the best way to learn how to perform surgery … obviously: is to do it yourself! (#22_41)*

*after all, our profession is based on the transmission of knowledge from older to younger generations, regardless of respective roles (#5_42)*

However, these interventions in the duo imply an intellectual and temporal availability of the trainer, calling upon his patience, and lengthening the operative programs.

*It’s difficult for trainer surgeons … you need a lot of patience, because you have to explain everything, say “no, not like that …, here, let me show you”, perform the procedure without actually doing it, as a demonstration, then hand over the instruments and let the trainee try. And all of this, well, of course it increases operating time by 1.5 or even twofold … but, if you are able to do it, then the trainee will leave thinking “wow, that was incredible!” (#11_27)*

These “ideal” teachers are then able to “let the trainee have their hand”, not taking over too soon, and accompanying them gradually.

*If you’re a bit too slow, don’t do the right gesture, or you don’t do exactly what they had in mind, etc, then they’ll take over. That’s something we don’t really like, we often talk about it amongst ourselves …: we’ll say, “they took over …, it’s a shame because it started out well, but then they took over for one reason or another” (#11_25)*

#### 3.5.2 Ideal sequence to progress

The ideal sequence seems to be observation/action/re-observation/re-action with debriefing of the senior.

*It’s important to let trainees operate, because even if you go back to just being an observer afterwards, if you’ve already done the procedure once yourself, you’ll pay attention to different things, you won’t focus on the same gestures because you’re trying to transpose what’s being done by someone else to your own hand, which is completely different (#12_34)*

Even after repeated passive observation and multiple corrections by their senior, they highlight the difference between observation and practice, as the conceptualization of the gesture can only be done by acting.

*Well, uh … there’s a big difference between theory and practice (#3_50)*

*There are times when … for example, some technical issue comes up that you hadn’t anticipated, and even though you’ve seen it being done hundreds of times, you haven’t conceptualised it, so you don’t really understand how to do it … so I think it’s good to have both [theory and practice] (#16_54)*

*I think first you have to understand what they are doing, why they’re doing it, in which order … why at that specific stage of the operation and not earlier or later, and then you have to actually do it yourself. And you have to do it with a critical eye watching over your shoulder, you need someone to correct you, especially when it comes to your posture, shoulders, etc the height of the operating table, eye height, with or without glasses, you need to have someone correcting absolutely everything you do, and then you have to do it on your own. It can be pretty tough. (#10_52)*

#### 3.5.3 Remote supervision

An essential step is to operate with the senior not immediately present in the proximity yet being available to avoid endangering the patient.

*It’s also useful to have to tackle certain issues on your own, because when there’s always someone behind you telling you what to do, well … you don’t actually ask yourself the right questions and … you don’t try to solve the issue on your own (#9_59)*

This situation stimulates the trainee's autonomous decision making, even if it is destabilizing.

*It’s good when you’re given the option … because it gives you a chance to assess whether you feel ready to take the leap and do it on your own, without a safety blanket, knowing that your supervisor is just upstairs in their office … or, you might realise that you don’t actually feel confident enough just yet … and you would rather they stayed nearby! (#21_62)*

*my mentor didn’t come in one day- not out of laziness- but because he knew I was capable of doing it on my own but that I just needed a little push to take on more responsibility. That was one of the first major leaps in my progress as a trainee, it was a bit unnerving at the time, but … well, it was enriching … it’s a good thing in the end because it helps you gain confidence (#13_63)*

#### 3.5.4 Key role of on-call duty

The time spent on call is experienced as a period where the trainee's autonomy is increased and is considered by all respondents as a primordial learning step.

*You can’t expect to be put on-call as a senior surgeon if you haven’t done lots of on-call duty as a junior surgeon first. (#15_69)*

*I felt like my level of skills wasn’t as much of a concern in the daytime as it was at 3 o’clock in the morning (#5_72)*

This autonomy in the technical gestures and in managing the different stages of a patient's care is the main approach for developing young surgeons into responsible professionals, while keeping a connection with the senior surgeon to prevent any failure.

*You do get given quite a lot of autonomy, whether it’s for indications, care, guidance, you’re basically on the front line, so you have to be able to work in a very autonomous manner … (#22_70)*

*You’re not left entirely to your own devices when you’re on call, but you are given a certain degree of autonomy to think, which is good, because the senior surgeon isn’t there to pre-process the work for you and tell you what to do for each patient. Usually, you get the call, you see the patient, and then you’re the one who contacts your senior after consideration to suggest a course of action- so in that sense, on-call duty is really beneficial for the learning process (#13_68)*

#### 3.5.5 Training between trainees

This progression also includes practical training by the older trainees, who participate in teaching, for basic procedures such as ventricular drainage or chronic subdural hematoma, providing valuable hands-on training.

*Actually, when it comes to the more … basic procedures like draining … it’s not the senior surgeons who teach us, it’s the older trainees … so transmission of knowledge really starts with our slightly older colleagues … and in any case residencies are all about helping each other (#19_30)*

##### 3.5.5.1 Stress of becoming a surgeon

Many respondents verbalize an apprehension about becoming a senior and a teacher themselves soon, even if the accomplishment of a successful procedure remains a great motivator.

*To think that in a year and a half I’ll be a senior surgeon working on-call at the hospital … well, I just don’t feel like I’m ready for that level of autonomy yet … for now, at least, who knows … maybe I will have progressed drastically by then … but, yeah … at the moment I don’t feel like I’ll be ready to work autonomously as a senior surgeon in a year and half. (#21_78)*

*The first time you perform surgery on your own and it goes well … yeah … you get in the lift, and you think “OK, OK, that was just … wow!” I think ego … ego is important in our field of work. (#21_82)*

However, being a surgeon entail more than simply the technical gesture, and for the trainee, this perspective is accompanied by an introspection on his transformation into a responsible practitioner.

*When you start out you don’t know how to do anything, but gradually you become more and more autonomous, you start giving your opinion, performing certain procedures, and you think ah, now I can manage, I am finally getting closer to becoming … well, a fully-fledged staff neurosurgeon … (#5_81)*

## 4 Discussion

### 4.1 Trainee-senior dyad

The training of future neurosurgeons involves interaction between the stakeholders of a dyad, and each of the two types of stakeholders has a facilitating and a resisting pole:

•The trainee, whose typology ranges from the one who is system-aware, accepting to be a team member and following the strict rules of the university hospital, to the one more individualistic (perhaps an echo of the millennials' generation?), seeking quick access to technical training without enduring the traditional servitudes, at the risk of disobliging the seniors and the team responsible for his/her training (Louie et al., 2019; Sarpong et al., 2022)
•Similarly, the typology of seniors ranges from the profile of the “ideal expert” who will devote time, patience, and energy to teaching step-by-step surgery to the younger ones to the one who does not help, who does not “give the hand”, who does not know how to, or who does not wish to delegate (Chikwe et al., 2004; Neal et al., 2022).

The respondents do not deny the existence of elective affinities that play a primordial role in the creation of trust essential to the delegation of tasks, despite criticizing the faculty who “don't know how to teach” (or who do not wish to devote time to the training of the juniors) (Svensson et al., 2009; Sterkenburg et al., 2010). While highlighting the personalities of faculties “who do not help” (Chikwe et al., 2004; Kelly et al., 2021; Vu et al., 2020), they also criticize their peers who are too demanding and who do not respect the rules of progression in their training (Bell, 2009; Evans et al., 2021).

The respondents immediately identified the value of working as a team, which goes beyond the operating room and encompasses the entire department and all the stakeholders involved in patient care (Veazey Brooks and Bosk, 2012). Similarly, they also realized that surgery is more than simply a symbolic technical act; it involves overall patient care, including the inevitable administrative elements and the relationship with relatives and families (Sterkenburg et al., 2010). In this, they acknowledge that their training aims to “make them neurosurgeons” rather than only “teaching them neurosurgery” (Jensen et al., 2018).

### 4.2 Heterogeneity of training according to the university centers

A majority of respondents mentioned the great heterogeneity in the training, among different academic centers with major theoretical and practical variations. The traditional model of surgical training is non-standardized and lacks individualism. That said, proposals have evolved since Halsted's seminal model (Kerr and O'Leary, 1999), and many studies depict attempts to harmonize neurosurgical curricula and possible custom adaptations to the personalities and learning abilities of everyone (Lipsman et al., 2017). However, so far, the training centers in our national territory have marked differences between them, dependent on interregional university managers, despite the marked efforts of the College of Neurosurgery (Campus de Neurochirurgie). We can hope that recent reforms will lead to a better standardization of the theoretical corpus taught to trainees who also attend European or international courses (Farges et al., 2020). Beyond theoretical teaching, university centers provide very different access to surgical autonomy, with structures that impose very early on the intern to operate alone at the risk of stress and reckless risk-taking, or on the contrary, that authorize very late autonomy, which generates obvious frustration (Jensen et al., 2018; Yaow et al., 2020). Finally, in a field where simulation has only recently been introduced, it should be noted that our respondents hardly ever mention it and that its use has not yet become standardized within training centers (Chan et al., 2021; Stengel et al., 2022).

### 4.3 Learning the hard way

Health anthropologists have aptly dissected the surgical ethos, legacy of macho heroes, virile and fighting values, professional rigor, and permanent availability (Cassell, 1987, Pringle, 2000, Yaow et al., 2020; Chan et al., 2021; Stengel et al., 2022). Although times are changing and the relationship between authority and hierarchy is being questioned even in neurosurgery (Majmundar et al., 2021), respondents report autocratic relationships with their superiors, negative interactions with authority and a reluctance to assert themselves in front of the hierarchy. These elements are described in many specialties and in all countries (Bongiovanni et al., 2015; Kamali and Illing, 2018; Ott et al., 2018; Lee et al., 2019). Even if the means of evaluation and the areas of improvement required of trainers are theoretically conceivable (Dickinson et al., 2020), the practical application is complicated, and the lack of training or excessive pressure exerted on trainees remains a problem that leads to unfavorable interactions between the different actors and even to the abandonment of a career at its beginning (Agarwal et al., 2018a). A framework for evaluation of the training system should be implemented, which presents practical and diplomatic challenges, in a system under increasing workload (Lipsman et al., 2017; Yaeger et al., 2020; Linzey et al., 2019).

### 4.4 Impact on personal life

The most influential factors in the appearance of symptoms of burnout or even thoughts of abandoning a professional project in surgery were the poor balance between personal and private life due to lack of sleep, major professional pressure, excessive working hours, and a poor relationship with the hierarchy (Louie et al., 2019; Shakir et al., 2018; Baumgarten et al., 2020). In our study, interestingly, the respondents complained more about the qualitative aspect of their work than the quantitative one and would be ready to work for extended hours if the quality of the work is worth it. The administrative workload is a major factor in the deterioration of the quality of their professional lives by reducing their theoretical and practical teaching hours and altering their professional satisfaction (Thomas, 2004; Mackel et al., 2021; Sadati et al., 2021; Rouxel et al., 2023). Once again, solutions can be identified, but their implementation is difficult. Regarding the administrative workload, there is a consensus that a decline in the work quality within university hospital structures imposes on trainees a difficult-to-return-to shift in tasks (Veazey Brooks and Bosk, 2012; Baumgarten et al., 2020; Woolhandler and Himmelstein, 2014). Formalized mentoring may be a satisfactory solution (Akhigbe et al., 2017; Singh et al., 2022), but in our country there is no formalized mentoring framework: our trainees almost never mentioned it.

### 4.5 Adult learning and surgical training

Surgical education follows the adult learning principles of integrating the learner into active participation in the process and identifying the learner's areas of development, including skills, knowledge, and gaps (Cope et al., 2017; Park et al., 2010; Mcleod, 2011). Historically, surgeons have integrated a professional learning technique that combines observation and practice of attitudes, gestures, and values witnessed by the juniors (Kieu et al., 2015). In the operating room, it is up to the teacher to find the balance between safe autonomy and active assistance, using objectives involving the trainee at each step (Lee et al., 2019; Stefanidis and Heniford, 1960), and insisting on a detailed postoperative debriefing (Roberts et al., 2009; van de Ridder et al., 2008; Bienstock et al., 2007).

Even if the time spent in the operating room and the volume of cases operated on are key elements in the acquisition of surgical skills (Birkmeyer et al., 2003), optimization could come from deliberate practice by setting adapted objectives, evolving performance standards, and conducting simulations (Kotsis and Chung, 2013; Ericsson, 2008).

In practice, the surgical training process is intensive, under the pressure of the chronic lack of time and availability of all stakeholders (Linzey et al., 2019; Stitham, 1991), and frequent stress and criticisms do not fit with reinforcement and positive feedback (Roberts et al., 2009). Therefore, maintaining a positive learning environment is necessary to support the trainee's ability to integrate and retain information (Kotsis and Chung, 2013; Usatine et al., 1997). The shift seems necessary toward training models based on individual competencies (not pure linear seniority) and the generalization of evaluation based on observable outcomes (Sterkenburg et al., 2010; Evans et al., 2021; Sandhu et al., 2017).

### 4.6 Empowerment

Regulation of working hours, changes in academic programs, medical-economic pressure, and the judicialization of medicine are the elements that impact the practical training of interns and decrease their early access to empowerment (Hashimoto et al., 2016; Chen et al., 2017). Several studies highlight the increasing lack of self-confidence of trainees to perform surgical procedures independently (Bucholz et al., 1960), highlighting a wide range between an early, insecure accession to surgical autonomy and, contrarily, a frustrating and equally insecure late accession to surgical practice. As illustrated by Hashimoto et al. it is an arduous but necessary task for teachers to create a new “See More, Do More, Teach More” model (Hashimoto et al., 2016).

Similarly, a significant percentage of surgical trainees feel that their training does not adequately prepare them to deal with the challenging situations they will face as a senior (Gkiousias, 2021; Coleman et al., 2013). Some studies have demonstrated the quantitative decrease in procedures performed by surgeons in training: pure casuistry is a paramount aspect of trainees’ training as well as a strong indicator of their proficiency (Agarwal et al., 2018b; Bingmer et al., 2020). And our respondents denounce both the administrative workload that prevents them from going to the OR and denounce the senior staff who do not let them “have a hand” during surgeries. Interestingly, the learning objectives may differ between trainees and seniors because the level of autonomy granted during an intervention seems more important to the senior than that perceived by the trainee (Hashimoto et al., 2016). Additionally, trainees expect to learn primarily technical acts during the procedure, whereas teachers are more interested in teaching them a global concept of decision-making (Levinson et al., 2010; Meyerson et al., 2014). Training also needs to be integrated within institutions subject to severe medical and economic pressures, with decreasing staffing levels and overcrowded schedules, particularly in university hospital centers where surgeons are also researchers and teachers (Stienen et al., 2018). This leads to conflicts with the time available for education in the OR, with the senior at the beginning of the day having a busy schedule to accomplish and at the end of the day still having many aspects of his professional activities to finalize outside the OR, the result being a lesser availability for training (Baumgarten et al., 2020; Young et al., 2017; Purdy et al., 2022). As in all countries, the laws imposing a reduction of the time spent at work have upset the training of surgical trainees as well as the functioning of the institutions (Bowyer et al., 2021). This obligation of compensatory time off has brought very positive elements to the global quality of life of the trainees, as attested by several studies (Baumgarten et al., 2020). Nevertheless, it is an additional constraint in squaring the circle of succeeding in training quality operators, clinicians capable of sharing complex medical decisions, synergic members of a multidisciplinary team, and eventually high-level researchers (Bernstein et al., 2006; Akhigbe et al., 2017; Cope et al., 2017). Currently, it seems difficult to achieve this ambitious goal without the standardization of theoretical programs, without a national reflection like what has been achieved in other countries (Lipsman et al., 2017; Stengel et al., 2022; Whitfield et al., 2023; Calderon et al., 2022; Henderson et al., 2020; Garba et al., 2022), and without the integration of new technological approaches in the training of young neurosurgeons (Stengel et al., 2022; Siler et al., 2023).

The French College of Neurosurgery has taken up this issue in accordance with the reform of the specialization cycle of medical studies, with the standardization of training at the national level being one of the objectives (Arrêté du, 2017). The College has defined detailed objectives and the means to evaluate them. National training days, local and interregional sessions are mandatory in the trainees' curriculum. And a campus-website compiles numerous online resources, enables the support of trainees, and also offers additional training, such as practical training, simulation workshops, focusing on a specific theme for two days, and using organic (anatomical dissection) or non-organic technical or behavioral simulation (Campus de Neurochirurgie).

### 4.7 Millennials or not?

In all specialties, decision-makers and trainers must cope with a generational gap regarding a “millennial” generation (born between 1981 and 1996, also known as Generation Y, which qualifies all of our respondents), and caricatured as spoiled children, individualistic, disrespectful of hierarchy, demanding, and wanting to limit their workload and schedule (Louie et al., 2019; Majmundar et al., 2021). Millennials, however, are described as both self-interested and altruistic (Desy et al., 2017), demand to know the relevance of the task assigned to them, and enjoy teamwork and collaboration (Williams et al., 2017). Millennials favor a flat infrastructure over the vertical organization of previous generations, which promotes collaboration, cognitive diversity, collective leadership, and autonomy (Eckleberry-Hunt and Tucciarone, 2011). Analysis of responses of our respondents reveals a more contrasting landscape, with millennial trainees criticizing the poorly distributed authority, work overload, or advocating the need for reduced hours or reform of a highly heterogeneous and perfectible training system, while also having perfectly integrated the hospital-university hierarchy and criticizing their peers who do not play the game of the good trainee who will submissively climb the steps of a surgical team one by one.

### 4.8 Limitations

Learning the structures and outcomes of a qualitative study can be disconcerting, especially for surgeon readers accustomed to statistically stated quantitative results (Starks and Brown Trinidad, 2007). Although the qualitative approach used in this study enables formulation of the hypotheses, it is not intended to validate them.

We interviewed trainees in the second part of their training so that they would already have sufficient hindsight to analyze their background. Moreover, we sampled as many respondents and academic centers as possible to ensure a representative sweep of the group of trainees in training nationwide during the study period.

We specifically describe the experience of young surgeons in training in our national system; however, we believe that their feedbacks and experiences are similar to those of their colleagues from other countries and medical-educational systems. Therefore, the expression of their feelings remains transposable (Jensen et al., 2018).

## 5 Conclusion

The perspectives expressed by surgical trainees may help understand their aspiration for improved educational assistance, both theoretically and practically, in the operating room. They emphasize the heterogeneity of their education both within their hospital and between centers at the national level.

The primary interaction with senior surgeons is at the center of their preoccupation with gaining mastery of the surgical act, which remains a subject of concern when they become independent physicians. The onset of independence in the operating room varies greatly amongst university centers, reflecting the heterogeneous nature of the theoretical training provided.

The conditions of training and progression are experienced as challenging in a harsh and unhelpful environment, and the training is often described as rough; however, the trainees are willing to put in a massive workload and endure intense conditions if there are qualitative returns.

The critical analysis of the strengths and weaknesses of their training and the formalization of their stress to become autonomous seniors can be a crucial link with the reforms and optimizations currently being made by the French College to enhance and standardize the training of young neurosurgeons.

## Declaration of competing interest

The authors declare that they have no known competing financial interests or personal relationships that could have appeared to influence the work reported in this paper.

The authors declare the following financial interests/personal relationships which may be considered as potential competing interests: None.
