# Supplementary material for: Becoming a neurosurgeon in France: A qualitative study from the trainees’ perspective
Source: Brain Spine. 2023 Sep 29;3:102674. doi: 10.1016/j.bas.2023.102674 (PMC10668099; doi:10.1016/j.bas.2023.102674)
Supplement: Appendix_File.pdf [file mmc1.pdf]

The Hard Way

In pain

And that's how you learn because you learn in uh... unfortunately in the pain and in the... in the hard way (#E1)

The beaten children

to hear him say certain things when he didn't do it either, it's not always very pleasant and that's what, uh.... I think, uh, damages certain, uh, interprofessional relationships because some people forget that they were residents, forget that they were in our place and, uh, consider that... uh.... That their word is golden and uh... and yours is not golden at all. (#E1)

We'll say OK you were on duty yesterday, well he's not here, he's fragile, and we'll move on, it's a little remark because people because they didn't have that... they didn't have that "right" well they say how come I didn't have it uh... while the little kid he has. So there's this pseudo little "resentment" that's there, so honestly if you have to... if you want to leave, no one will tell you no, you can't leave. I mean, it's never happened to me. (#E11)

Bad atmosphere - workload

Well, in fact, it was a period of uh... we did three shifts... one shift every three days, there were only three residents on the duty roster, so that made ten shifts per me, it's very tiring and then I managed thirty patients, which is a lot even for an old resident, I was young at the time, so on top of that, she yelled at me almost every day, it was really uh...(#5)

Survival at the beginning...

But the beginning of the residency was essentially survival, I think that unfortunately there are residents who live like that throughout their residency, it's that survival, that is to say, day to day, managing to go to the operating room and uh... and not kill anyone, that was just that. In the beginning it was really uh... being there every morning, trying not to kill patients and trying to cover all the blocks of all the chiefs because we... we would get yelled at if they were alone without an resident. (#E10)

The right to say no

Uh... on the training of surgeon... ah yes I think that... something that is important to say, is that we... we don't have much right to say no. (#E10)

It's really ingrained, nobody ever says no. So I don't say no to little things, I mean from home I do the consultancy uh... I organize social stuff, I take whatever I'm asked to do, but I did say no once and frankly it took him a good seven or eight months to get over it, to talk to me again. (#E10)

The world of Care Bears

when you do something stupid, you get scolded, it's normal, when you do well, they don't necessarily tell you that you've done well, but that's... that's how it is, it's an environment, it's... that's a bit what I was saying about the. ... the atmosphere of this specialty is that we are, um... a somewhat tough specialty and so, of course, we... it's not the world of Care Bears where we say bravo, it's good what you did, things like that. We only point the finger at the negative things in general and we don't necessarily point the finger at the positive things, but we're used to that and we... it's not a suffering, it's just the way it is, it's a fact and so uh... so personally I don't have any relationship problems. (#E11)

No compliments

Afterwards, the surgeon will say uh... once again because it's not a milieu that is very uh... that is very much into helping uh... "To put it simply, no boss has ever told me that you're too strong, you have superb hands, etc., uh... they'll only tell you when you've done something wrong... they've never told me uh... ah bravo, that's great. On the other hand, when you've done something stupid, they'll tell you it's bad, so to come back to that, it's hard to make up your mind, but uh... so uh... I think it's the same for everyone. (#E11)

The unspoken rule

and then well... there is a kind of... rules in surgery that say that the more... you have to be there all the time, never be tired, and so on, show that you are there, don't take a rest,... that's from the old generation and that it's time for that to change and it is changing, which is very good. (#E12)

General atmosphere

If we were allowed to operate a lot during our residency, that I saw a lot, well the young surgeons let the residents operate a lot, if we were not allowed to train at all during the residency, uh... the young people tend not to let the residents do anything at all, uh... if there is a department that puts a terrible atmosphere, a terrible pressure and that you can get uh... you can get smashed in front of everyone at any time, well it's not the same atmosphere (#E12)

the goal is to pull people up and make the younger ones want to do it and not uh... not to take them apart to see uh... who will resist and who will be above to be wary of what. (#E12)

The handyman

I understand that it's uh... that it's very difficult to live... to have the impression of being the handyman because clearly I think that uh... it's a bit the image that... that we can imagine of the resident, it's yeah the resident is a bit uh. ... the one who is going to be in the thick of things, who is going to be everywhere, who is going to do everything, the tasks that are a bit thankless... well yes, on a daily basis, that's a bit like that, but uh... after all, that's how I perceived it, if we play the game, well, we are rewarded, there you go

Difficult position of the resident

is that people, even patients, understand that the resident is a doctor. So of course he's not a doctor, he's not a doctor, but he's a doctor and as such he has credibility so uh... and the chiefs they have to accept it too, it's not because uh... it's uh... the resident that uh... he doesn't know uh... There are some people who assume that even if an resident has a good answer, it will still be stupid because he is an resident, so if... I think that if anything needs to be improved, it's that, (#E13)

Assignment in the specialty

we don't teach pawns, we teach people who have ways of doing things and ways of learning, and in fact, no one takes care of that, and I think that the resident, uh of the first semester, he was completely broken because he was pushed into something that he was not able to manage, he was too autonomous, too empty, too alone and he had to say to himself that the specialization was not made for him whereas if he had been taught in another way, maybe it would have happened differently, but that's a much broader thing. ... much more vast, uh... that we won't be able to change just at the local level, it's the way we assign residents to the specialties, (#E14)

Human dust

the idea is to not look at the... the residents as numbers and right now we're just numbers so uh... [Name X] was ranked 215, he was the first, we took the first, he chose... well result... result uh... human dust...(#E14)

Tolerate...

otherwise I... well I knew that it was going to be very, very hard so uh... the first year I may have uh... tolerated things that I thought were normal and then as time went by I... well I said to myself it's going to be super hard, you'll have to... in surgery you have to put up with everything, etc. (#E2)

Horrible first year

I did a first year where I... where I left at midnight, came back in the morning at seven o'clock, I was already in the hospital because everything had to be perfect, uh... everything had to be managed on the first day, uh... in relation to... in relation to the contact with the nurses, with the department managers, uh... with some surgeons as well, how they... well, respect for the resident, doing a proper job without being under constant stress all the time, that's what the first year was like for me... well... during the first year I didn't realize it, but now I... I know that I had a pretty horrible first year. (#E2)

A first-class asshole

You have to know how to take yourself in hand and you have to... you have to be able to say to yourself, uh... it's okay, uh... he's a first-class asshole, uh... so what he says doesn't get to you, and uh... there you go, but they're not all like that. (#E6)

Getting yelled at at night

now that I don't dare anymore because I know that if I got yelled at, it wasn't because I woke him up, but because my clinical examination was incomplete, because I didn't know what to do, because... because everything was complicated at the time (#E10)

Getting into the program

I found that in France it's either you do... globally it's either you do or you don't do... in any case in my training I'm talking about, I don't know how it is in other cities, but I had the impression that most of the time you... especially at the beginning of the residency you are an operating assistant so you help, you help, you help because you have to go fast, you have to get the operation done uh... the surgeon is in a hurry because he has a lot of operations and uh... the operating room has to close at six o'clock, the last patient has to be operated on so it has to be finished, etc.., so it has to be done quickly, so of course speed means that the resident has... he takes more time so we don't have time to let him do it so overall we do a lot of closures at the beginning of the residency, we close uh... the tissues, the different steps and the more we advance, the more we close uh...(#E11)

Young seniors

In fact, chief of clinic means that it's uh... it's someone who has finished his residency... well, who has just finished his residency so uh... it's a bit of a pompous title to be honest uh... that doesn't mean much, but chief of clinic means that it's a guy who has just finished his residency and who has passed his thesis and so on... and who is supposed to train residents above all, but he can't train residents when he himself has not been trained. (#E6)

The golden word

to hear him say certain things when he didn't do it either, it's not always very pleasant and that's what, uh.... I think, uh, damages certain, uh, interprofessional relations because some people forget that they were residents, forget that they were in our place and, uh, consider that, uh.... That their word is golden and uh... and yours is not golden at all. (#E1)

The pathological personalities

it's true that surgery is... it's an environment where there are a lot of obsessive, of... people who are not very patient, people who want things to go quickly as they want, so if you operate a little too slowly or if you don't do the right thing or you don't do exactly what he had in mind, etc., he's going to take you back (#E11)

Secretary

Oh yeah, yeah, clearly. Well, in fact we do... in fact... we do all the work of the secretaries except for taking appointments, so um... so yeah, the administrative task is absolutely enormous and um... and on the one hand it's not training and on the other hand um... it's time-consuming and... and at the end of the day it's... it's time wasted doing something else. (#E6)

Administrative constraints

For the "old chiefs" who did their residency twenty years ago, which had nothing to do with the current residency, they seem to say that it was worse for them because there were fewer residents because there was more work, etc., but on the other hand, I don't think that it's a good idea to have to deal with a lot of paperwork, but on the other hand, I think that it is difficult to compare because there are many things that have changed, particularly in terms of administration, information, etc. Now we have a huge amount of administrative and computer work compared to what we had before, when, for example, many prescriptions were given orally, but everything has to be traced, passed through the software, signed, multi-signed, uh... even the smallest things, removal of a redon, urinary catheter, I don't know what, all the reports have to be complete, uh... sent to all the correspondents, done on the day of discharge, uh... and uh... detailed, etc., so uh... there are a lot of things like that that uh... I think have changed...(#E15)

we had an residency that wasn't comparable and maybe they're having a hard time realizing that workload and in any case that... uh... a lot is expected of us anyway (#E15)

we have an inadequate prescription software that makes us lose hours to do the smallest thing, so I think that .... we easily lose an hour to an hour and a half a day on the uh... on the prescription waiting time, but uh... but on the really paper side uh... report, etc., there is not so much stuff... (#E16)

it still takes up a good part of the day... between the prescriptions, uh... the letters, the trajectories, uh... all that, uh... yeah, it's still a good part of the day. The proof, for example, is that I left my "on-call rest" late, um... I'm not obliged to stay in the department, we're perfectly entitled to our on-call rests, but here I am staying to catch up on all these administrative tasks that I wasn't able to do, um... under normal circumstances. (#E20)

Strained relations with the team

the beginning of the residency was very complicated with the residents who were above me, who behaved... in retrospect, like a bully... (#E16)
the newcomer who dares to have an opinion, uh... and dares to open up even though he's new, well, that didn't go down well at first, especially with the young people, in fact, the old chiefs don't care, but uh... but the young clinical chiefs and the old residents, it was very complicated with them, yeah. (#E16)

for me in medicine from the first year we are taught to crush ourselves, to shut our mouths and... and to endure and me to have done something else and to have come back to the hospital after uh... I knew a normal process so when I have a problem I say it and then when I don't agree I say it and I argue it and uh... so it makes a bit of waves and... and it's not well perceived. (#E16)

Quality of life

In terms of quality of life then on the other hand, I was maybe uh... I knew it was a very difficult specificity, but uh... we don't have the best quality of life, but I maybe didn't expect uh... to make so many sacrifices. (#E15)

Finding your place

I could have said the absence of... how to say... framing at least what you're supposed to do as an resident. I arrived, I had to learn what I was supposed to do on the job and no matter how many times you ask the question "what am I supposed to do", the answer is "you'll see" and then after that it's learning by uh... it's learning on your own what your job is as an resident, beyond surgery... how you function in the hospital, your place in the team uh... (#E16)

A stooge

I don't have decades of experience, but uh... there are things that can shock or things like that where uh... the lack of confidence of... of some surgeons and what makes it... it's horrible. It's horrible the way they relate to you, the way they talk to you... the way they look at you.... Not as a future colleague, but just as a stooge... (#E18)

System not adapted

I think it's because the system is not adapted, there are too few of us, but at the same time, uh... but at the same time, you have to be, uh... you have to be confronted with many... a lot of cases to be uh... to be trained and that only happens if we are there every day. (#E19)

Abandoned

we need a little more support. In any case, the feeling I have is that I'm a little bit, uh... a little bit, uh... not abandoned, but here's the thing, uh... if you want to come to the OR, you come, uh... but no one supervises it... it's not supervised in any case, it's... so you end up learning on the job because you're there every day... (#E20)

Bad boss

He said to me "well listen uh... I hope you have courage because you're going to go home every night crying at one o'clock in the morning and uh... and you'd better be pretty serious because otherwise there's no point in crying and at least make it worthwhile uh... because it's going to be uh..."... Okay... we started off on a very bad footing from the beginning and then it was... it was someone who was quite unpleasant, uh... quite unpleasant and then it's a person who doesn't necessarily have confidence in himself and who compensated for that with a lot of aggressiveness in fact. (#E20)

Lack of time

the department uh... well, from what I could see anyway, the surgical departments are managed by the residents and not by the chiefs, so uh... well, there are forty patients so we have to take care of them every day and it's up to us to do it, so it takes uh... it takes quite a bit of time and energy to do all the things that we have to do. and energy to do all the entries properly, ... Uh... so keeping it up to date is necessary, but it's... well, it takes a lot of time in fact and... and you have all the computerized prescriptions, all the reports, all the discharges, the discharge papers. I think we have a major administrative workload and paperwork (#E21)

The joy of the residency

I think I always did what I was asked to do, even the boring stuff. Honestly, sometimes I do things while complaining, but I do them. After that, that's the joy of boarding school, there's still a lot of boring stuff, that's for sure, but... in the end you get used to it. (#E23)

Hard places

I think that there are services in France, if a boss meets you at five o'clock in the afternoon, you're going to be dismantled, you see... I find that stupid too. Because if you have nothing to do, uh... you're in the hospital to do nothing, there's not much point. So uh... I chose [City B] for that, so after uh... I know uh... I know the cities where I shouldn't have gone and I didn't take them, it's true that they're cities that uh... that have a bad reputation because they force the residents uh... they. ... they don't respect all the conditions; they abuse a little bit uh... the guys are there from 7 a.m. to 11 p.m. every day... my opinion is a little bit biased because I chose Bordeaux for uh... for this kind of quality of boarding. (#E23)

alteration of the desire

I wasn't at the bottom of the bucket, but I felt that I was a little fed up because I didn't really want to be in the hospital anymore... so it was very temporary, but in fact what I realized at that moment was that it wasn't the quantity of work that was bothering me, it was my quality of work (#E23)

The shift was cool because we kept operating, I was happy, I saw stylish things and the chief was there on duty, it pissed him off, but in the end we got out of the OR, he ordered something nice to eat, we ate together, we laughed um... you know it was cool and... so this is my personal opinion, but I think that the... the problem of burnout I think is the quality and not the quantity of work and I would much rather have three shifts and work seventy hours, but with chiefs who are cool and with whom I get along well than to do forty hours, but to be bored, to be yelled at, to do things that are visits, not many blocks and things that bore me uh. ... or doing a block and it doesn't go well, I'm not happy with the result... it's... it's a small aside, but... in any case the two or three times I was fed up were really that. (#E23)

Easement of the structure

The old habit

there are many things that are not part of our job that we do every day, but that didn't shock me because I had seen that as an extern, uh... stretchering, uh... making various administrative requests for home care workers, doing social work, doing... well, we were used to that as an extern, so it didn't shock me more than that. (#E10)

Discharges

it's the discharges that are extremely constraining because it's fighting against the whole world to find places for patients and having the impression that there's a shift in tasks at the university hospital, that is to say that some of the professions at the university hospital willingly leave some of the more thankless tasks to the residents. So that's what's a bit annoying, it's when you feel like you're doing someone else's job (#E13)

The job of the physiotherapist

Yes, so basically it's a pain in the ass for that, it's that there are certain things where we do the job of social work, uh... we do the job of a physiotherapist, we do... so it's a bit of a pain in the ass, so administratively it's a pain in the ass. (#E13)

Obstacle

Maybe we haven't talked enough about... the problem of the structure in fact which is the hospital because uh... I think that this is one of the... the hospital is... its functioning is an obstacle to the practice of medicine and also an obstacle to... to the training of residents because, uh... there are a lot of administrative requirements and a lot of tasks that... uh... that compromise the proper functioning of practice (#E5)

How would the hospital run without the residents?

And these are things that fall on the residents and that push back training a bit and we have the impression that finally we wonder how the hospital would run if the residents were not there and ... we say to ourselves that uh... we are supposed to be here to train, we want to be of service to the patients first, to our team of uh... of... of neurosurgery in our department, because if they pass on things to us, we are also quite ready to... to give... to give service to the team, to the structure, but we have the impression that... that the hospital behind it uh... and the health system a little abuses our situation what... we are a workforce uh... qualified enough to do a certain number of things uh... at quite advantageous costs. (#E5)

Obstacle to becoming a surgeon

That's one thing that's a bit of an obstacle to the quality of our training because it takes us time that we can't allocate to researching, reading, and writing. to do research, to read our books, to prepare our blocks uh... I think that this is one of the obstacles to... to our training to... to become uh... to become a surgeon. (#E5)

The adjustment variable

we feel that it's... the training of residents in fact is... it's the adjustment variable in our function in fact. (#E5)

The hospital is running less well

obviously we don't pretend to be indispensable but there are... times when we don't come to do the... uh... the task... well, to fulfill our role... to do the tasks that are a little bit ungrateful, well, the hospital runs a little bit less well and so finally we can't prioritize our uh... our training. (#E5)

What is the responsibility of the resident

Uh... well, it's secretarial work, it's uh... it's acting as a nurse or checking that uh... the guy's not in a coma because the nurses don't know how to score a GCS correctly, uh... it's uh... acting as a stretcher-bearer to take the patient to the scanner, it's uh... well, it's a whole bunch of things that don't necessarily fall under the resident's function, but that the resident does all the same. (#E6)

It's part of the duty

you never go to the operating room... well, it's exceptional to go to the operating room and say to yourself I don't want to uh... it's... it's really the thing that we like and on the other hand everything else can be a bit painful to go and see the families, these are moments that are not necessarily pleasant, we do it because it's our duty, but it's not something that... We do it because it's our duty, but it's not something that... "that makes you feel good", it's something... it's moments that aren't necessarily very pleasant, not always, so there you go, it's part of the duty, but it's not what we like the most (#E11)

Secretary

In [City P], it's really the “secretarization” of... of the residents, (#E14)

On the other hand, it's rather as time went by that I realized that I was clearly doing things that were not part of the residency and I would say that at the beginning of my residency I didn't have that feeling, but the more time goes by and the more I... the more I really have that feeling (#E6).

Those who don't help

At the end the disappointment

so there you have it, finally, at each block it was the ultimate stress and uh... at the end the disappointment, finally, there you have it, so it was... blocks that were quite difficult to live through. (#E2)

Those who don't help

in neurosurgery, we can do some procedures, uh... well, some surgeries "on our own", uh... simple surgeries and emergency surgeries, so, uh... there is both this place that, uh... uh... that... that we quickly take, uh... on the on the first operator's shifts, but as far as the other surgeries are concerned, it's a bit senior-dependent, that is to say that with certain seniors we... we quickly get the hang of things, others will let us do less (#E4)

Manage yourself alone

I see for example in [City T] uh... we know the people of [City T] well because they are part of our inter-region, ah well in [City T] on the other hand it's crazy. In [City T] it's uh... yeah well you're going to learn surgery, well you have to do it, but well you're all alone because uh... the boss has two rooms running at the same time, that is to say two patients. Well, the boss doesn't have the gift of ubiquity, so he can't operate on two patients at the same time, so it's the resident who does it, but the resident is all alone, so I think that's dangerous for young residents. (#E13)

Territorial heterogeneity

The resident chooses his city and his department under the impression that he understands how things work and then he will find himself either in a department like mine, which is very permissive, where he is very autonomous very quickly, or in another department, I don't have an example in mind, but I would say [City P], where you don't do anything and you don't touch anything until the end of your fifth, sixth or seventh semester, and there is no regulation...

Here better than elsewhere

The training for us, well, to... to plagiarize the words of a boss, well, yes, it's not much use, to... to go and see, the most important thing is to... to learn what we do here. Well, I think it's a lack of... of open-mindedness because uh... after... we are formatted... we only do what we have been taught locally and uh... we don't learn other techniques or things like that. (#E3)

New concepts
I learned a lot when I moved to the spine unit and uh... it's true that there are some concepts that are uh... that are quite new... well that are uh... that are less than ten years old what about sagittal balance in particular, etc.., and uh... that we don't necessarily apply uh... in the departments where we uh... that are called cranial let's say and I find that quite a pity because we have learned things uh... in the same hospital and in fact we realize that uh... well, depending on the affinity of the head in question to put in screws or not, we do it more or less, whereas potentially, according to what is described in the literature, there is a real indication. So I find that a little bit, uh... apart from the fact that it's a pity from a point of view and from a conceptual point of view, it's a pity for the patient because it means a loss of chance, either in terms of spinal pain, or in terms of... well, after all, it depends on what we're talking about... what we're talking about, but uh... but I think it's a shame, yeah. (#E6)

I don't want to let go
And with the old surgeons and the young ones, but the problem is that the young ones don't want to let go of everything either because they want to practice so uh... so they keep their hands on many things (#E1)

In your corner
If you don't get along with your practitioner, well, it's just... they're people with whom you don't have a connection at all, um... it's going to be cold, it's going to be distant, um... he'll do his own thing and we'll try to help him as best we can even if we don't like each other at all and we're not here to like each other either, we're here to uh... to work together but sometimes it's not always easy and we mustn't forget that we're also human and that uh... that... that the working relationship will... be also based on a personal appreciation that can vary this work atmosphere and this is also very important because the more you get along, the more you will do and the less you will get along and the less you will see and the less you will practice with this surgeon (#E1)

The young and the old
So there are surgeons who often find themselves left out of... of residents because uh... because no one gets along with them or because they don't let anything happen or because uh... it's... it's over like that and it's people with whom we don't uh... we don't enjoy working and failing that there

are young residents. ... there are young assistants who have just come out of the residency and who are there to uh... to operate on anything that moves because it's also uh... at that point that we can get uh... our, our medals(#E1)

Those we don't go with
Yes, yes clearly. Learning, recognizing the steps, uh... then starting to do a step during the surgery and then progressively doing more and more, yeah... yes... and then there are surgeons who are... who are ultimately very educational and uh... who... for example on a small metastasis... well, he'll tell me I'm the operating assistant, you're the surgeon and who's going to be there to help, to hold, to spread, uh... to cut the sutures and to guide me if I get stuck, uh... on certain steps: in the end, we sort things out, we go more with these surgeons than with the other surgeons who don't let us do much. (#E1)

The suture maniac
in the end, I do four sutures in the whole surgery, in the fourth semester of my residency, he will cut two of them because they are not perfect, so there is no... we can't learn in the end because he lets us do absolutely nothing, we're always just there cutting the wires, watering We're always just there to cut the wires, to water, so... with him, there are practically no residents who go to the OR with him. (E2)

The beaten children
it was also a bit historical... we have chiefs who are generally quite old, um... and they, well, they had an residency where the chief didn't do anything, so they reproduce a bit what they saw. (#E3)

Not very pedagogical
so I told her, "No, I can't do it alone. He didn't really like it and in the end he did his patient like a big boy who didn't need me, he wasn't very... very educational, he could have shown me once and told me that afterwards you'll do it on your own, it didn't happen like that, (#E14)

The old school
when I started the residency, uh... my former department head was a bit old school, he never moved, we found ourselves as young residents doing things alone at night, etc. That changed a lot, uh... (#E4)

2 hours later

... because being all alone in a room when our chief... the chief who arrives two hours later, uh... I think that learning is null because in any case we do as uh... well as we are uh... a little obliged to feel like doing what so uh... so there is no learning uh... well it's not like that that we learn what. I mean, I think it's a form of learning, but I don't think it's a good form of learning, at least not at first. (E5)

The U of University

we'll say the... the reproach I have for them is that they are not pedagogical enough... so on the practical level, they don't give us enough gestures during surgery and on the uh... on the theoretical level, they don't do a lot of uh... in the end, they don't give staff lectures, they don't... not to do any theoretical teaching (#E15)

We forget a little about the fact that it's the university hospital because it's there to teach. (#E15)

A real involvement in training in the sense that most of the Pus (University Professor) uh... never give courses, do nothing and uh... and one wonders why they have the U of U in the history uh... what I was going to say uh... well yes in fact it's the setting up of a structured curriculum.(#E16)

Give and take

We have one or two who are in the mode "yeah I'm here to operate, I'm staying" uh... the chiefs don't respect them, young and old alike uh... so when they ask to... once in a while they ask to do something on uh. ... on a rest day, well, as for me, I just came off duty today, I was asked to replace my boss at the consultation, so uh... well, I told her no and so I know that I'll be seen as a slacker again (#E16)

Insufficient theoretical training

At the local level we have some kind of biblio' sessions, but they are not courses, but they are made by the residents, given by the residents with chiefs who are only young clinical chiefs, so people who don't necessarily have much more hindsight than... especially me now that I'm an old resident uh... and so in my opinion they don't bring much to the question and once again it's courses that are based exactly on who to operate on, but never on the how of the why uh... we go about it what.(#E16)

Personal shortcomings

me being totally unable, unlike some of my co-residents, to get home and open a neuroscience book, uh... well, uh... I learn on the job, it's quick, so it's okay, but uh... after uh... well, afterwards it's fine, but uh... but if they wanted to give me a theoretical neurosurgery exam at the end of my residency like they do in other countries, uh... clearly uh... it would be complicated, but that's usually true for most French people. (#E16)

Heterogeneity of the Centers
it's really uh... ultra dependent on the resident himself so for me uh... well here we can't all be identical robots and each one has his strengths and weaknesses, but there we... we don't know in fact... we don't know... what we want an resident to know how to do at the end of his residency and clearly, even if within a city it tends to be more or less homogeneous, in any case uh... I think by knowing uh... [City M], [City T] and [City N] uh... how we work, but I'm sure that uh... well I already know that an resident from [City T] cannot do the same thing as an resident from [City M] at the end of his residency, I'm sure that he can't do the same thing as an resident from [City P] either and so in fact we have people who arrive at the end of their residency in France who have very disparate levels. (#E16)

in the north they have structured courses over five days with a program established four years in advance so everyone is certain to cover all the aspects of neurosurgery, there are sites like [City G] where they never have any courses at all. Here, um... we have one course per semester and often the course is organized ten days beforehand, including for the speakers. (#E16)

It's never structured... well there's maybe one or two places per exception where it's structured, but uh... the learning... the follow-up of the technical learning not being structured and not having clear objectives on what an resident is supposed to be able to do, there's no follow-up on the. ... the residency and so uh... we also arrive at a very disparate level uh... of residents and that's why I went to [City M], I was told "if you go to [City M], you will know how to operate at the end of your residency". (#E16)

Alignment of the planets
I thought it was going to be more of a uh... coaching uh... something where you have a mentor. But we talked about it last time, but it's a... it's a bit of a roller coaster, that is to say that you get along well with someone, uh... there's no second surgeon uh... he's got time behind him, he's not in a hurry, he doesn't have thirty-six thousand operations, he doesn't have to leave so he lets go of his hand to. ... to do a small part and in fact it's necessary... it's necessary for the planets to align for it to go well, whereas it's not a systematic will where there's really a notion of progression, of objectives and everything. Not at all, uh, supervised... (#E18)

Heterogeneous motivation

There are some leaders who really have the fibre, who really have the desire and there are some... it's the least of their worries. Then I think they are tired, that is to say that uh... when you have a ten year career and this is the twentieth class of residents and that uh... well there is a moment, if the resident doesn't have the right uh... the right attitude or I don't know or whatever the reason is or you're not uh... you're not well on a personal level, one can imagine that you don't want to train at all. (#E18)

Those who help... and the others

it's clear that it's... already dependent on... of the boss with whom you work, there are bosses who in any case, whatever happens, whether you're competent, whether you prove yourself or not, will never let you do it and there are some with whom uh... the current passes well and who are also open to train people and those are uh. ... so they're more open to uh... to letting us take on more and more responsibility uh... but on the other hand.... even on interventions that they know that... that you're capable of doing, there are some leaders who absolutely won't let us do it. (#E19)

Those who do not help

well, there are a thousand reasons, it could be, uh, lack of time, or, uh, wanting to do the procedure perfectly as we are used to doing it, or it could be because of interests, uh, frankly, I have many reasons, there are some chiefs who, even if they are experienced, even if they are old, even if they have done the same procedure two thousand times, still won't let us do it (#E19)

Those who do not help (bis)

I have quickly uh... put myself to... on these interventions in a systematic way and he let me do more and more and it's clear that the chiefs who didn't let me do it and who wouldn't let me do it, well I turned away from their block because I knew, out of interest, during the uh... the duration of the residency I... there was no reason for me to go and look at things if it was for uh... not learning anything or not... or not getting my hands dirty (#E19)

They did not see me

the leaders who didn't want to, uh, train me or let me have a hand in it, well, they didn't see me. That's how it went... quite easily. (#E19)

Appetence to the transmission of knowledge

It's about doing your job well, but a little bit in all the... in all the facets of the multifunction and once again, with some leaders it's not enough and then those, uh... I don't really know them. I get along well and I do what they tell me, but I don't go to the OR with them. My strategy was to target the chiefs who had a little appetite for transmitting knowledge (#E19).

Lack of theoretical training (heterogeneity of the territory)
we are registered at the university, we are supposed to be students, we have zero courses so the theoretical training is exclusively at our expense, one hundred percent. If you don't want to learn, if you don't want to learn, you don't learn, but I think it's a problem. That is to say, uh... we should have courses and evaluations and controls, but... we have neither courses nor evaluations, I think that... in a university training it's stupid, but that uh... in neurosurgery it won't change because the chiefs are... the chiefs are too busy, they have... they have too much work to do, uh... the courses are of interest to only a... a very small proportion of... of the teachers. (#E19)

Lack of senior practical training
... No one cares if you're uh... autonomous on this gesture at this time of the residency uh... (#E20)

The one who sleeps at night
I didn't really know how to do it and I found myself in the operating room with the chiefs who were snoozing in the middle of the night and who didn't want to come, even though they knew very well that I wasn't very comfortable... so at the time I wasn't very serene, eh, after the moment when you're an resident, um. ... you're not responsible for much, eh... afterwards I try to do things well but well... if I do something stupid and the guy is at home, I don't have much to lose... (#E23)

Progressive autonomy

Verbatim+++

I remain convinced that it is the resident who makes the quality of his residency. (#E13)

but I remain convinced that it is the resident's investment that will make the quality of the residency, it's... basically it's a question of return on investment (#E13)

We don't arrive with the same basis

You have to understand that we don't arrive with the same foundations even if our training is theoretically similar, we don't arrive with the same foundations at all (#E3)

Late start

And it's perhaps on this that... that I regret, it's the simple gestures of training that... that train us a lot because when we're young the slightest thing... makes us dream a little because that's how it is and we like our job and that... that's how we imagine it, but it's sometimes difficult to have to wait two - three years before... to use a Kerison forceps and to know in which direction you hold it.(E15)

So, apart from seeing it, we only learn very late to make gestures that we would have liked to intellectualize more gradually than brutally over a hundred meters (#E1).

At the beginning was the observation

at the very beginning when obviously we are in the first semester, we are really there to observe, we learn the basics, to know how to close, so uh... the place is more that of an operating assistant who observes (#E5)

Team building

So more autonomy... empowerment uh... well I think it's mainly through integration. In fact, if there's one word for it, it's integration (#E11)

Positive effect of the lack of personnel

There were only two seniors left in the department and so two physicians in the department to run the department that I'm in now is uh... it's really very hard. So they really relied on the residents who were there and it's true that during that semester I really exploded surgically, they really let me do a lot of things, they taught me a lot of things, they trusted me and it was really a give and take, it was really uh... you do things well and there's no reason why I shouldn't let you do things, whether in the OR or in the ward ward (#E6)

Not daring

At the beginning of the residency because uh... it's particular, I was the only neurosurgery resident so I was doing shifts with chiefs who were used to doing shifts with old residents, I was yelled at all the time, all the time because I... I didn't have the experience... it was inherent to my age, that's it and they were sleeping at home and uh... and yes it already happened, yes. It happened that I put patients in danger because of that... now that I don't dare because I know that if I was yelled at, it wasn't because I woke him up, but because my clinical examination was incomplete, because I didn't know what to do, because... because everything was complicated back then (#E10)

Autonomy too fast

just the same, the heads, um... they're still professors over fifty years old who are tired of running, um... the store, um... I think, and there weren't enough young heads, so, um... there wasn't a lot of mentoring, and, um... there was a little more, but it's a bit of a vicious circle, that is to say, when you're in your first and second semesters, we let you do a lot of things, well, we have the impression that in the third semester you are already very old and so, uh, we spend less time, uh, teaching you things because we have the impression that you already know how to do everything by yourself, which is not true. It's a... it's a subject uh... that we talk about every day on the ward with the co-residents, (#E14)

Not a helpful bystander at all

So we have... we are evolving towards a progressive autonomy. At the beginning, we were, uh... we were considered as, uh... more or less a spectator, a... not at all useful but uh... to become the being uh... because at the beginning we don't know uh... at all to install a patient, we don't know what our chief uh... more particularly is going to expect since the expectations vary according to the... the seniors. Well, in a few years at most, we'll be in his place as a senior, so we have to gradually replace him, while not necessarily having the best possible training in what he would like us to be able to do. (#E1)

Initial uselessness

I expected to do uh... a lot more surgery than... I did at the beginning, I expected to operate more at the time uh... with age I realize I wasn't capable of it so fortunately I wasn't operating more. (#E10)

Temporal progression
for our leaders it's very difficult to have us at the beginning when we are clumsy and we do anything we have to be uh... very careful about what we do uh... then there is a period when they are happy that we help them and then things are going well and then at the difficult period they have to teach us things, they have to be, they have to have confidence in us and that actually that... that's done in stages. (#E10)

First we have to prove that we are not dangerous, then we have to prove that we are useful, uh... and then we have to prove that we have surgical potential.(#E10)

Finishing the residency
I'm exhausted, but I've done as much surgery as I can because my biggest fear is to finish my residency and not know how to operate. That's something that terrifies me so uh... I operate all the time, all the time (#E10)

Self-doubt
I was very... very brave at the beginning, I was very proud to have operated on someone by myself at night, uh... and now we... now it's good, but uh... I'm not sure if it's uh. I'm not sure if it's the best thing for the patient... some operations where I'm stuck calling my boss in the middle of the operation because I can't do it, I still think it's more dangerous for him than being operated on by my boss (#E10)

Next year...
at the beginning of my residency, it was rather positive, that is to say that I was impressed with what I was allowed to do in retrospect, with my current point of view, and I said to myself now if I had to... since next year I will be chief of clinic, I will perhaps have young residents like me do things and I say to myself that I would not have allowed them to do that, (#E11)

Not in danger
I didn't feel like I was in danger once in my residency on a procedure that I didn't master or uh...when I was allowed to do it, it was either simple procedures, or that uh...I knew how to do because I had seen them, I had already done them with chiefs, etc., so I felt like I could do it on my own;

or most of the time the... the chief surgeons were there and so uh... the procedures were done under supervision and so if it got too complicated or there was a problem, the hand would be taken over by the... the chief and so in that case it would go on as it should normally go. (#E11)

Drop the hand quickly
I didn't expect... I didn't expect to have easy access to the OR because it's the reputation of the neurosurgery resident, neurosurgery residents have less access to the OR than other residents and finally it was quite quick in the training, I think it was quite progressive and we were let go quite quickly and in an adapted way (#E12)

Companionship
you start by learning to open, then you learn to close, uh... with... with... during the first years with a chef present and then progressively once they know us uh... a chef present in the room, but more dressed, then a chef who is not present in the room and who comes to see uh... so there is really something very progressive uh...so there is really something very progressive uh... well very chef dependent anyway uh... very access on the companionship (#E12)

Early stage
Uh... my place in the OR... uh... I would say uh... the first six months I didn't do much, uh... it's observation, the second half of the year we really start to get our bearings and to be comfortable with uh... sterile dressing, uh... well we start to know... to understand, let's say, all the gestures, um... they start to let us close because the first things... in the OR, the first things they let the resident do is to close and they start to let us open a little bit too, the second thing they let us do (#E12)

Progression through the emergency room
afterwards we are allowed to do quite a bit, but uh... especially in the emergency room, the emergency room shifts, so emergency skulls, emergency spines uh... do everything that is opening, closing of skulls, evacuation of hematoma uh. ... laminectomy for the spine, removing the posterior part of the vertebrae, etc. and, uh... yeah, they start to trust us to, uh... let us close, leave, leave us alone in the room, etc. (#E12)

Access to complex procedures
I would say that it's from the third year onwards that we start to let go more and that the surgical resident becomes... really starts to become more experienced, to do more in the OR, um... so in the third year we start to put in screws, we start to... to remove tumors uh... well I'm also lucky to

be in a department in [City T] where uh... I did half of my semesters so they know me well, so they let me... I think they let me go more than in another center because I always come back to the same place (#E12)

Surgery from A to Z

the surgery from A to Z, I would say that it's the third year the first time even if there's always a chief who's watching, who's there on the side, but to really let them do the elective surgery and to really start to manipulate it well is the third year. Then the fourth year, uh... I think we start to have a little more autonomy over it, uh... yeah I would say... uh... my last year, I left (#E12)

you are the one who operates and you present your file to the staff, saying "here I have Mr. what's-his-name," and you have your operating indication validated by the staff, and then you find yourself... and that's where the difference lies, you find yourself a chief to supervise you and... and operate with you (#E14).

then I feel like we... maybe I'm left to think more about what we're doing, the planning, the setup, trying to... to know what the next step is going to be and gradually I'm allowed to do parts of the surgery, but very gradually and it really depends on which surgeon I'm working with (#E2)

Overview of the residency

Basically I said that the first few years they let us uh... well, the first year was the beginning, we started to do the small gestures. In the second year, we started to do all the procedures, uh... emergency surgery, but always with someone. In the third year we were allowed to do more of the uh... scheduled surgeries, that is to say non-emergency surgeries in the usual neurosurgery days, that is to say everything that is a brain tumor, uh... we started to put in screws, to do narrow lumbar canals, herniated discs, uh... well, we did more and more uh... and from the fourth year onwards, uh. ... it's still rare that they don't let us do a block... well I would say that at least half of our blocks they let us do... and after the fifth year, in my opinion, that's when the resident... in any case that's my case, um... we start to try to train in a different specialty, that is to say to invest more in one of the fields. (#E12)

Natural progression

In fact, it's quite simple, uh... well, I was lucky enough to have worked in the private sector as an operating assistant before starting my residency, so I was, in fact, uh... basically quite biased in the OR and in instrumentation, so, in fact, uh... I naturally took my place as the first operating assistant, the place that an resident has, uh. ... that, that was... in fact it's done by itself the first times and then I think that it's what allows the chief

to judge a little bit uh... the way we understand surgery ... then progressively well I went uh... from first operating assistant to... to first operator on certain times of the surgery in a uh... way uh... in fact it was very repetitive, it was for such and such a surgery, I was in charge of doing uh... such and such a part uh... basically uh... I was in charge of the exposure times of a tumor for example, and then, depending on the difficulties and the increasing level, I took more place in the procedures, in the totality of the procedures, but it happened quite naturally. (#E13)

Responsibility
yes, I am autonomous in my decisions, but... so I am comfortable, but uh... I don't feel completely uh... surgeon for the moment because I think I haven't really been confronted with the job by having the responsibility alone. So technically I think that uh... yes I have the surgical logic, but uh... in fact it's just a leap in the dark actually the... the last day of my residency and the first day of my clinic there's nothing that's going to change, it's just the responsibilities and I think that's just what's extremely scary and makes me doubt the fact that... well I'm going to be a surgeon soon and not quite. (#E13)

Progressive technical training
Afterwards, um... I find that in fact we have a technical training that is very good, um... that is very progressive and that respects the... the level and the limits of the residents, that is to say that we are not thrown into doing anything on our own and at some point we don't feel restricted either, saying to ourselves, um... ah well, I would like to operate, um... that's it. (#E13)

Non-linear progress
it's a bit of a vicious circle, i.e. when you're in your first and second semesters : we let you do a lot of things, well, we have the impression that in the third semester you are already very old and so, um, we spend less time, um, teaching you things, because we have the impression that you are already very old teaching you things because we have the impression that you already know how to do everything by yourself, which is not true. It's a... it's a topic that we, uh... we talk about every day on the ward with the co-residents, (#E14)

I see the progression
there's more of this huge stress in my head of... I'm too young, I don't know how to do it, I'm too young, I don't know how to do it, I'm all alone... uh... it's... oh well it's going well, it's cool uh... here I've finished my thing, I'm going to call him, he's going to come and see and then you've got the PU who comes and completes, who continues or just checks and closes, so yes, that I... I see progress. (#E14)

Take action
finally start to let us do it because I find that... even if we see several times... well, I have trouble even if I see the surgery twenty times, uh... the first time I'm going to be left to do it myself, I'm going to ask myself a lot of questions that I've never asked myself, uh. ... because in the end, when the chiefs do it, we have the impression that it's super easy, that it's logical, that it's always the next logical step... but when we have to do it ourselves, well, we ask ourselves a lot of questions and so it's good to... to try to do, (#E2)

Even if we watch a surgery several times, uh... the day when it's our turn to do it, we ask ourselves questions that we had never asked ourselves before, so yeah, we let ourselves go more in the OR (#E2)

Theory and practice
So uh... well theory is one thing, practice is another. (#E3)

Progression
So it evolved from the test phase which lasts a little month to uh... quickly bah so we start the surgeries uh... in partnership with the surgeons and then we... we close very quickly afterwards. That is to say, we close... well... without the need for a surgeon and then after the phase between opening and closing, well, that's as we... we give you more and more control (#E3)

So we go from second operator to uh... closer and second operator during the phase... to first operator and then first solo operator (#E3)

Degree of empowerment
I think you have to leave the... the local decision as they wanted to do for uh... the degree of empowerment uh... because uh... each resident is not the same and each structure is not the same so it's a little bit complicated to... uh... to generalize the thing knowing that it's a rather... important period, it's the period I'm living now uh... since my fourth year uh... and uh... indeed it really depends on... on the person. There are people in other centers who... they don't get there so easily so uh... you have to... well, you have to... you have to... the autonomy is not so abrupt because otherwise it can really put uh... put some people in... in positions that are not pleasant at all and...there are patients... there are patients in the middle anyway (#E3)

From A to Z

as we go along, of course, we can... we are the main operator with the help of the chief, who is there to supervise us a little, or even to take over if we are in a difficult situation, or if we have difficulty doing part of the operation, and then... ... then we can be completely autonomous, that is to say that the chief knows that we are doing the intervention obviously, but he is not necessarily in the room and we do the intervention from A to Z all by ourselves, so uh... here is the autonomy uh... it's... it varies according to the interventions we do and the... the evolution of our residency as well. (#E5)

Patient choice
So that's another parameter, even if the emergency will be taken into account, they won't let us do uh... let us take, sorry, the same responsibilities for uh... an elderly patient who is in a cranial state uh... well a serious clinical state with a relatively simple intervention uh. ... on the other hand, if it's a patient who is uh... young uh... who is neurologically well, who has a prognosis that is uh... that is excellent if he is uh... well operated, well maybe they will be less inclined to let us do it, especially if we haven't done this type of operation before. (#E5)

As the years of medicine go by
this feeling comes and goes in fact because uh... in fact as you go through... of all the years of medicine like that, because in fact you realize... so when you arrive you don't know how to do anything and then little by little you become autonomous, you start to give advice, to do this or that type of intervention and you say to yourself ah here I am capable of managing, so you say to yourself that finally you are getting a little closer to... uh... of what... well, of an attending neurosurgeon...(#E5)

Team Dependent
Yes, yes, uh... at the beginning there are quite a few uh... observation phases, uh... in fact I have the impression that it's a little bit uh... in stages, eh, it's uh... you stagnate for a long time and then you increase suddenly and then you rest and then uh. ... and then I hope that it's good... well I have the impression that it's a little uh... it's binary uh... but in the long run, it's uh... it's stagnation for a long time and then we have .... I feel like it can get unstuck a little bit like that overnight and I think, again, it's really dependent on the team, it's uh... the team... it's the willingness of the guy that's there to train you. (#E6)

Frustration / Progression
It took a few months, so at the beginning I was a bit frustrated and then, as time went by, it was uh... we were allowed to close, then we were allowed to make some gestures, there was also more... more autonomous initiative that I didn't take at all before, but now that we know a little bit

about uh. ... well the heads and the rest of the operations, well we can anticipate the movements and we also try to arrange the head uh... the stupid things, but if we do a tumor removal, we hold the tumor for him a little bit, things that I would never have done at the very beginning of the residency. (#E7)

I'm given a lot of freedom

on the surgical level, uh... uh... something like that which I didn't necessarily expect and which I find good, is that they let us have a lot of freedom... well, they let us do a lot of things ourselves by supervising us, by explaining things to us, etc., which was less the case in Nantes. They let me, I don't know, set up by myself, start by myself, they don't necessarily come and check what I've done, uh... there are certain interventions they let me do by myself from A to Z, maybe come and check what I've done at the end just to check, but... but without necessarily helping me to do it and they know that if I ever have a problem, I'll ask for help, that. ... well that's it... someone will be ready to come and help me uh... or vice versa whenever uh... I do it with someone, he lets me go ahead and then when he feels that I'm in difficulty he takes over, but that's how he did it from the beginning, they just do it later and later in the operation we'll say. (#E8)

Supervising

we always have someone to supervise us, but it's not necessarily someone who will do it for us. He'll look at how we do it or if it's... well I don't know if it's spinal surgery, if there's material to be put in place on both sides, for example I'll do one side and he'll do the other side and then he'll give me advice for uh... for my side what. At the beginning, he would do my side completely, then afterwards he would let me do a little bit, and then little by little, he would let me do it. (#E8)

Never alone

They can let me start on my own, they can let me do everything without getting dressed or anything, but they don't even come to see what I was doing, except for the chronic hematomas and for the external shunts, um... that's not... well, I'm thinking, but I don't remember. At least they'll be present in the walls or they'll come by to say hello at some point to see how things are going. (#E8)

Heterogeneous according to the centers

in fact I was quite surprised by the autonomy and the uh... the resourcefulness uh... from the first months in fact of the residency what... that's something that surprised me a little at the beginning uh... afterwards I find that with uh... the experience uh... the experience, one is quite easily uh... well, you get used to it quite easily and you adapt to it quite easily ... I recently did ... well, I'm currently doing ... an residency ... what we call

an “outside” residency, so ... an exchange in [City T] in another department ... after that, I think it's quite heterogeneous, depending on the ... depending on the department. I found that in my current residency, in another university hospital, there is less autonomy and you are less abandoned from the beginning of your residency. (#E9)

Progressive scheme
After a few months, so we'll say three to four months on really on the uh... basic gestures uh... we are autonomous so first with a senior who is uh... on the spot in the room and then quite quickly uh... with a senior who is uh... present in the hospital and who is not necessarily in the uh... in the intervention room and so there we are led to do the procedure alone and uh... if ever there is a problem or we have questions, we call the senior so that he comes to uh... to look at us or to help us. (#E9)

Start with the end
well, little by little we start to do more and more... more and more procedures and then some steps uh... so quite easily at the beginning what they let us do is the closing uh... that's the step after the surgical gesture itself, so we do the closing and then uh... after uh... little by little we can do the opening and then the closing uh... and then after a part of the gesture uh... under supervision and then little by little uh... sometimes all the gesture depending on the interventions (#E9)

Importance of being solo
afterwards, I think it's... it's also good to be confronted with certain problems because when you always have someone behind your back telling you what to do at what moment, in fact you don't... you don't really ask yourself the questions and you... you don't think for yourself about finding a solution uh... so I think it's good to have a little bit of the... the different ways of... to function in order to be trained. (#E9)

to spot the traps
well, it's something that comes quite gradually, but uh... it's something that's more recent, we'll say... from the end of my third year and the beginning of my fourth year when uh... when I'm asked... well for example when I have the telephone advice, I'm asked for uh... for something uh... I have the impression yes bah to know what to do uh... in what time frame uh... well to know how to organize things uh... to... to spot the different traps (#E9)

From A to Z

if the operation goes well, we can manage the surgical procedure from A to Z, we manage the post-op procedures, etc., the... and so we actually... we do the whole... the whole chain of care from the preoperative consultation to the operation, to the postoperative consultation, we are uh... we are almost autonomous uh... supervised if necessary by the senior, so uh... yeah it's... it's autonomy, uh... where we do the activity of a surgeon, uh... senior under supervision, but uh... but basically if it goes well and it's something simple, uh... he hardly intervenes in the... in the process. (E23)

Evolution of the ability to manage problems
we feel as we go along that the... we are more comfortable in the operating room, we have more... technical abilities and above all, uh... something that I think is important, is uh... knowing how to adapt to the... difficulties that we encounter little by little, but that's something that we have thanks to experience in fact, it's after having seen uh. ... problems arise and it's with the management of these problems that you learn to manage it yourself and uh... I find that this is really what shows the... the... the... empowerment and then the... uh... well we'll say the evolution of the uh... surgical capacities. (#E9)

Never in danger
It's something that I have the impression was quite fluid and... and progressive in fact during... during my residency uh... where you are confronted little by little to more and more uh... more and more complicated situations uh... but I was never in any case uh... I never felt in danger or uh... abandoned on uh... on a situation where I was in difficulty, so that I think it's very important uh... but globally I think that it, it goes rather uh...(#E9)

Secure progress
After uh... I find that in fact we have a technical training which is very good uh... which is very progressive and which respects the... the level and the limits of the residents, that is to say that we are not thrown into doing anything alone and at a given moment we don't feel restricted either by saying uh... ah well I would like to operate uh... there you go. (#E13)

Temporal progress
In fact nothing is... nothing is harmless, there are things I always realize... they do things, they don't tell me why and I realize very late that in fact everything has a meaning and that... we don't see it right away so I think first of all we have to understand what they are doing, why they are doing it, in what sense uh... why at a given moment of the operation and not later or earlier and then we have to do. And then you have to do uh... with a critical eye over your shoulder, you have to be corrected, especially on the posture, on the shoulders uh... on the... the height of the table, the height

of the eyes, the glasses, no glasses, you have to be corrected on everything, everything, absolutely everything and then you have to do it all alone. Great moment of solitude. (#E10)

To revise the anatomy
I'm not ashamed to say that some surgeries, even today, I still review the anatomy a bit before going to do something to get it back in my head, not to be surprised, so I think that I learned well by doing... by rereading a bit of anatomy before, but especially, especially by going to see what it was in real life. That's it (#E13)

A middle ground
he tells me "listen, you start, I'm coming". Well... what do you mean... yes, yes, you start, you set it up and then uh... you make the incision, you put the carotid artery on the side and you move forward and then you put the carotid artery on the side, during my third week of residency, the world was already falling on my head, I was no longer with my friends, I had left my city... well, it was all nonsense and he said to me, "Put your carotid artery on the side and move forward... I won't forget that one...". After that it's true... it's a bit the opposite extreme of some services and a middle ground with a bit more support it could... it could be nice. (#E14)

An important step
in fact, we are on call, it's a tandem chief, uh... resident, the resident is on site in the hospital and the chief is on call at home, so he travels, uh... if need be, uh... and in fact he didn't stay at home out of... I'm going to use the word... out of laziness, he stayed at home because he knew I was capable of it and he wanted to push me around a little bit. ... to shake me up by making me take responsibility, so that was one of the first steps at the beginning of my residency, which was a little destabilizing at the time, but uh... it's rich, uh... in fact it's good in the end, it's a good way to gain self-confidence, that's all. (#E13)

transpose the gesture
I learned best when I was left to do it, so uh... after having observed several times, I think it's important to let go of the hand because even if afterwards you spend several surgeries being an observer again, already the fact of having done it just once, well you don't look at the same things anymore, you don't look at the same gestures anymore, you're more interested in the same things because you try to transpose the gesture of the other to your own hand, so it's still very different (#E12)

See, do and review
I think that... in order to learn a gesture well, you have to see it a little, for example see it once, twice, do it with someone who guides you by saying "no, that's not good" and then see it again... well... I think you have to alternate between seeing, doing, seeing, doing because uh... I think that when you try to do it, when you realize that it's complicated... because when you see someone doing it, it's super easy, but it's not super easy (#E8)

well I know I've thought about it a lot on skull surgeries, tumors or things like that where they let me do more and more, I tried to do it, but it seemed much less easy to me than when I see my chiefs do it and then I saw where I was having trouble and I saw them do it again afterwards and the next time, well there are things where I had made progress on my gesture, but I need to see them do it again and try again...(#E8)

View again after having done
And I realize that it brings me a lot to see someone else do it once I've been left to do it, for example for surgeries... I had noticed that for herniated disc surgeries, at the beginning I couldn't do it at all, but now I can do it... on my own when it's... when it's not a herniated disc that's uh... hyper complicated or other, when it's a classic herniated disc we'll say, but uh... little by little by watching people do it, I realized that this way of doing it was more efficient, that it worked better, (#E8)

Differential Empowerment
on the practical training uh... here it is a little oriented in the sense that we are taught very quickly to do uh... which will allow to relieve a little bit

... the chief during on-call duty, etc., but... on interventions that are perhaps programmed... where the chief does not necessarily need us to help him or do things for him... there we are much less empowered, much less... involved, I would say. (#E5)

Acting on it
Well, a ventriculocisternostomy, which is an operation, uh... well, my chief used to... well, it was an emergency block and my chief asks me, uh... before the block when we wash our hands if I've ever seen one. It's an operation that we see, but it's not very common, so I said yes, yes, I've seen two or three, but without any ulterior motive, he told me you wanted to do it... of course he was there, he helped me and everything, but... I wasn't expecting it at all. (#E7)

A little worried

we could discuss it for an hour, eh, about our training, but, uh, already I think it's a problem of... that is at the national level in fact... it's very good that we are autonomous in our training, it's a bit normal, we are adults after all, we've been hanging around at the university for several years already, so we're supposed to be able to train a bit on our own, but I'm still a bit worried (#E5)

Rather fluid
It's something that I feel has been quite fluid and... and progressive in fact during... during my residency uh... where you are confronted little by little with more and more uh... more and more complicated situations uh... but I never felt in any case uh... I never felt in danger or uh... abandoned on uh... on a situation where I was in difficulty, so that I think it is very important (#E9)

Surgical reasoning
Ah yes, maybe so... so create a kind of reasoning with a reflection because we sometimes don't think enough, in fact, we operate because we want to operate or because... well I say that when we are residents, I mean we want to operate because we want to operate, the chiefs afterwards know, that's the difference between the resident and the chief moreover, it's his operative indication that is much better, that's a little bit there. ... that's... that's the most important thing to remember for the residency and the practical side, but that's personal and so here's trying to stimulate reasoning and create it, it's... already so that the resident creates a surgical reasoning and thus chooses an operative indication (#E11)

Being a neurosurgeon
being a neurosurgeon is not just about, um... opening a patient's head, removing the tumor and closing the head, it's everything that goes on before and after, whether it's about... indications on the theoretical level or on the human aspect, how we manage, um... the announcements, the explanations of, um... the operating risks, etc. (#E5)

Surgery is not only the operating room, the operating room is a big part, it's probably the most important part, but it's also the management of patients before and after the operation, the relationship with the families, so the relationship with the nurses, the health care manager, the nurses' aides, the staff... (#E11)

Those who make the difference
generally speaking, we all arrive at the same level, we're all surgically equivalent over the years, depending on what we have to do, and then, afterwards, in my opinion, we stagnate until a certain semester, and afterwards, the difference is really those who work a little more than the others,

who are a little more present, who do things without being told, and who take the initiative, etc. Well, it's going to work well for them, no doubt about it. (#E12)

Too fast, too early

we don't teach pawns, we teach people who have ways of doing things and ways of learning, and in fact, no one takes care of that, and I think that the first semester resident, uh of the first semester, he was completely broken because he was pushed into something that he was not able to manage, he was too autonomous, too empty, too alone and he had to say to himself that the specialization was not made for him whereas if he had been taught in another way, maybe it would have happened differently, but that's a much broader thing. ... much more extensive, um... that we won't be able to change just at the local level, it's the way we assign residents to specialties, (#E14)

Extern to resident

I was surprised by the gap between the autonomy we have as externs and the autonomy we have as residents...we have the prescription uh...on all decision making of course uh...if we always refer to our...referent, to our senior, well we don't have any role anymore so we have to filter, we have to judge if it's urgent, not urgent, we have to see if it's in the field of neurosurgery or in the field of... of medicine, um... and so there are times when... for example we say "no, this is not a big deal, we'll see tomorrow" and um... it's still a very big responsibility (#E18)

Limited responsibility

even if in reality we don't really have any responsibility because we are always more or less protected by the... by the... the public hospital in the sense that... unless we make a big mistake or there is a real lack of will on our part... otherwise we are always protected by someone, either by the guy who is a senior, or by the hospital... (#E18)

Not ready yet

... when I imagine that in a year and a half I'm going to be a senior and do my shifts at the hospital, I don't feel that I have enough autonomy at the moment, maybe it's going to explode in the next year and a half, but I don't have enough autonomy at the moment to be able to do senior shifts in a year and a half. (#/2/1)

Progress on the notebook

In fact, in total, over uh... so I'm now at six and a half semesters, I'm uh... at 1021 surgeries, including uh... 501 cranial surgeries, 423 spinal surgeries and then the rest, well, other surgeries, and I think I counted the number of... uh... in fact I don't have the number of surgeons who are the first operator in the operating room, the first assistant, the second assistant, and overall I made a little graph and it's true that you can see little by little over the course of the... the semesters that the... the percentage of second assistants decreases, the percentage of first assistants also increases, and finally the percentage of first operators increases. So it's also indicative that there's a gradual, um... autonomy. (#E15)

A little too fast?
I did my first skull on my own in the third semester, with the chief behind the door, but uh... so no, they are aware, they let go of the hand rather quickly and then it remains a little personalized in the sense that every morning it's not the same... afterwards, sometimes in retrospect, I would say that we are let go of the hand perhaps a little too quickly. (#E16)

That's not what I thought when I was there I was very happy to... that they let me do a lot of things, but in retrospect, a few years later, I say to myself that uh... certainly I knew the gesture, but maybe not with enough uh... confidence to be able to uh... to face up to a problem or to be sure to do the right thing... (#E16)

2 modalities of progressiveness
So for me it's progressive for everyone uh... but then there are those where it's going to be progressive in the sense that the two I was talking about before it's progressive in the sense that we're with them and as our residency progresses they let us do... well, they let us start the intervention and they take over later and later, and then there are the others where they let us have progressive autonomy, so never when we are in the operating room with them, but in the complexity of the gestures that they will let us do, um... in total independence. (#E16)

I think it's not bad to have a combination of... the two modalities with us, that is to say, people who say to you "hold your scalpel like this, do it like this, cut it like this..." and others with whom you'll be watched more, but... by interpreting the autonomy that you are acquiring with others and then finding yourself alone with them... those who are next to you, who accompany you as you go along, never give you anything to do alone, whereas those with whom it's all or nothing, well... there are those moments of... solo where you find yourself... well, if there's a technical question that you hadn't asked yourself before, even if you've seen it 250 times, you hadn't conceptualized it, so in fact you don't realize it, and so juggling the two is pretty good (#E16)

Adapting to technical variations
"The problem is that, well, the problem is that, uh, it often happens on shifts and so each time we have a new shift, uh, as soon as it's with a new chief, he doesn't really know where we are personally, so he wants to show us his technique in relation to other techniques that vary according to the chiefs and the residents, so we'll say that, uh, after after three or four times, I started to do it with them, accompanied, well, I was the one who helped them, and they were on the side, and now I'm starting to do it after... after... well, four or five times like that to do it in front of someone, uh... I'll start to do it alone, uh... that's it (#E17)

Elementary gesture vs. global gesture

I really understand what we do now, uh... at the beginning, uh... on the blocks, we arrive and then we... well, we don't really know, uh... from A to Z what's going to happen during the operation. Now, uh... I know what's going on, uh... and so, uh... it's more, uh... and when I'm given instruments, uh... I'm much more, uh... how should I say it... I can handle them much more easily than before, and so, all of this, it... it helps to gain more self-confidence (#E17)

We are no longer, uh... focused on our gesture that we are doing, but more on the gesture, uh... well the global gesture, uh... that is being done during the operation by the chief and uh... well we are no longer focused on uh... our little hands and try to see not to tremble or to uh... well not to struggle to hold the instruments. to hold the instruments. (#E17)

Evolution towards autonomy
I find that my autonomy has increased a lot, even in the guards, not only in the operations, but also in the opinions, by dint of reviewing the same clinical cases and having the chiefs explain to us why we do this, why we do that, after a while we manage to answer much more easily (#E17)

The ideal scheme
The best way to learn with the chiefs would be to be with the same surgeon, to see once, to do once, supervised, once, twice, three times the same surgery, the same pathologies and to do another time without the guy in front of you, who is available, you know, because, uh. ... because when he's there and when he's not, well, um, I don't do the same thing, so that would be ideal, but it never happens, but, um, but, um, that would really be ideal (#E18)

Chronology
after the place in the OR, well, at the beginning anyway, uh, that is to say, during the first six months, uh, in the OR, we do nothing, well, we do nothing, I would say that, to be more detailed, I would say that for three months, we do absolutely nothing other than watch, and the next three months, they start letting us close the scars, and after the first six months, we start doing, uh, some, uh, we start operating, uh, a little more... what. They start to let us do the opening, to do the work of ... quite minor, but to manipulate the tools what... manipulate the instruments. (#E19)

Graduality
Afterwards, uh... I couldn't tell you exactly what stage happened and it's... it's gradual, it's... I worked most with uh... the same person who gradually trusted me more and more and then there was a moment when we knew that she could stand next to us and watch, eventually uh... guide the gesture, but to go into more detail than that, I... I'm sorry, but I think I would have difficulty. (#E19)

The blast

in Brest they let you get your hands on a lot of things very quickly, so that was... very satisfying because in the end that's really what it's all about, the... the... the joy of residency is touching the OR and here in [City B] you have access very quickly I think to... to be able to do things uh... quite quickly in autonomy. In fact, it's good and I expected that it would be a little later in my... in my training, that I would be able to be as autonomous on uh... on many things and in fact no, we were quickly autonomous so it was... nice. (#E19)

Adaptation to the mentor
there are some chiefs who like the residents to take the lead, to have everything set up, to make sure that when they arrive everything is OK, while there are others who want to supervise everything, so you have to adapt to the chief and if you manage to know how he wants it done, it often goes well (#E21)

Finding your place
At the beginning, well... you don't know where you are, you don't know where you're going... so it's really... it's... you... you just don't take too much initiative, you wait for someone to tell you to uh... you're uh... hyper attentive to what's going on, it's a lot of observations of everything, even of things, well... of... whether it's in the gestures, even in the relationships between people or uh... of the installation uh... of everything. Really the block is hyper codified in fact uh... everything is... it's a kind of choreography... it's always the same in a few things and so uh... you arrive and you

don't know... well you don't know the steps what. So it's complicated and then afterwards you start to understand better and better and so you manage to get into the thing, to get into the rhythm and uh... after a while well... there you go... when you start to do things on your own, you're the one who's going to give a little bit uh. ... the rhythm of it all, so you really go from being a complete observer to uh... to someone who will take real initiatives and who will... here's where you'll be listened to, all that, there's a pretty important progression I would say. (#E21)

Giving the choice

That's it, but it's good when they... it's good when they give you the choice because that way you can evaluate yourself a little bit and see if you want to... if you want to get past the stage of doing everything on your own without a net, knowing that he's in his office two floors above, or if you're not yet sure enough of yourself to uh... and you want him to be around a little bit. (#E21)

To pass a course

it's a step to go it alone without your boss with you, in fact I like bosses who ask you, you know, who will tell you uh... "so do you want me to be there, do you want me to be dressed, do you want me to be unclothed" and that's cool because you say to yourself well... when you don't feel ready yet, you say "well yes, I want you to be in the room" because you're not serene and then once you're comfortable you say "well no, it's okay, don't come uh... I'll send you a message when it's over uh... or I'll come to your office when it's over". (#E21)

Becoming a surgeon

I couldn't tell you exactly which OR but, but... I would always say first times, the first time you do a procedure by yourself and it goes well, well... yeah you go back up, you're in the elevator and you say to yourself "OK, OK, that was... wow! Ego... ego is important (#E21)

A generalist school

I think it's a very good school, uh, generalist, uh, where we are given a lot of control, I think, I don't know about the other centers, but, uh, I think a little more than the other centers, uh... so it's very good for everything that's basically learning the basic gestures, afterwards it's true that when you want to do more superspecialization, I think you have to go to... to centers that are a bit bigger, um... I did my inter-CHU in [City M] for a semester, it was more for everything that was base of the skull, etc, what. (#E22)

From the spine to the brain

we are autonomous in a rather progressive way, at least in , where we let you do the simple gestures and then progressively in the semesters that progress we let you do more things. So at the beginning we let you have more autonomy on the spine uh... because it's a little less uh... less delicate and then at the end of the residency we... we let you have access to the... a little more to the cranium and the brain uh... in a progressive way...(#E22)

Example of a time line
yeah in the first few weeks I was doing small external ventricular shunts or spikes uh... basically at... shortly before Christmas - we're back in November - so a month and a half, two months, uh... I was doing the... the chronic subdurals, uh... and then the first kyphoplasties, uh... kyphoplasty still under control uh... let's say, but the chronic subdurals I did in my corner as uh... a little bit as I wanted so uh... after I started to do the kyphoplasties uh... alone uh... I think uh... at the end of my first semester beginning of my second semester, I was doing everything alone in my corner uh... after it became what... after it was a little bit uh. ... basically on the shifts I was doing the extra-durals uh... all alone with my chief who was next to me, who was watching me, who was dressed, but who didn't touch anything uh... after that I started to do a little bit of spinal work as well with a chief who was next to me, he was doing his side, I was doing my side, that was the same for my second semester, I still had quite a bit of help huh. That was for my second semester, I still had a lot of help, and uh... from the third semester, uh... more like the fourth semester, I think, because I did a semester in neurology, I did a little bit of other things, uh... and then I started to do a little bit of skulls, uh... always with assistance, where I was left to, uh... to do my own thing. (#E22)

It's not so complicated, after a while
I find that it's very progressive in fact. At the beginning uh... at the beginning you grumble a little because you don't do anything, which is normal since you're not very good with your hands when you arrive and uh... at the beginning I said to myself "there, fuck that, I don't feel like doing it at all, it's the same" and then as you go along, with more and more repetitions, with more and more... with more and more going to the block, with more and more seeing things, you realize that it's not that complicated. (#E23)

To impose oneself in the team
I think that in the first and second semester I was also apprehensive about being a bit alone, more than necessarily about the gesture, the fact of being a bit alone in the room, uh... first operator, it's not easy you see when you arrive, you're twenty-three - twenty-four years old uh... managing the teams, getting them to listen to you when you're a first semester resident, you're going to tell me that the nurse who's been in the profession for forty years, uh... I think that... she doesn't even know your first name at the end of the semester... you don't have much clout, so that's... that's something that you learn too, how to manage teams, how to uh... everything is learned. (#E23)

I didn't really know how to do it and I found myself in the operating room with the chiefs who were snoozing in the middle of the night and who didn't want to come when they knew very well that I wasn't very comfortable... so at the time I wasn't very serene, eh, after the moment when you're an resident, um. ... you're not responsible for much, eh... afterwards I try to do things well but well... if I do something stupid and the guy is at home, I don't have much to lose... (#E23)

Eulogy of the repetition
I think that there is a big part of... there is a big part... because in fact technicality is the repetition of gestures, well anyone can do anything if he has seen it a hundred times. I'm learning the piano now, I didn't do it six months ago, now I can play music because I've learned by heart with my hands when to play which note, I'm not a pianist. I'm not a pianist. You just have to practice and you'll get it right. The question is... I think that uh... it's true that people who are rather anxious will find it much harder to be autonomous in the operating room and to manage the complications even if they are perhaps a little more gifted with their hands (#E23)

The key word is "progressive
the key words are progressive, it's really the key word of the residency for me is that in fact, uh... first, second semester, uh... sometimes I would say to myself "fuck it but uh... "I mean, when I saw surgeons struggling in the OR, second semester, I thought "damn, I've been an resident for ten months already, I can't see myself doing that, it looks unfeasible, it looks horrible", I was happy to be an resident in my corner watching rather than being the chief who is getting bored... (#E23)

The positive effect of bullshit
I've never had any serious adverse events, that is to say, uh... that prolong hospitalization, uh... that create damage, but I've done a lot of little things in the OR, uh... that didn't have any consequences, but it's by doing these little things that I learned. And I think it's also important to do stupid things if of course it... it's sure that if you kill the guy uh... yes it's not important to do it, but uh... but uh... but I think that doing little stupid things uh... technically it's very interesting. (#E23)

Tame your senior

No right or wrong way

well for me there is not necessarily a right or wrong way to... to learn, but I know that... well, in the surgeons we work with, we know that we are used to it and that we know that they won't all work the same way. (#E9)

Participate in the emotional charge

I remember that at the beginning he didn't talk at all, then we had a very, very difficult case, it was a very, very complicated moment of adversity and he realized that he could count on you because you were there, because you were managing things while he couldn't, because it was emotionally difficult and you were taking on the emotional burden of this case that had been evolving for more than a year. ... which has been evolving for more than six months and people are dying and it's really very complicated and there's a kind of click (#E10)

You've got it!

from that point on, things uh... things take a slow upward slope and then until uh... until you leave the hand in the operating room. It takes hours and hours of... of blocks together. I also think that they see us evolve every day and that at some point, uh, we help them well enough that they start to help us, but in any case with us, You've got it! (#E10)

Those who do better

generally speaking, we all manage to, uh... we're all surgically equivalent over the years, depending on what we have to do, uh... and then, uh... after that, uh... in my opinion, we stagnate until a certain semester, and after that, the difference is really those who work a little more than the others, who are a little more present, uh... who, uh... who go and do things without being told, and who take the initiative, etc. Well, it's going to work well for them, no doubt about it. (#E12)

Keeping your distance

I'm not uh... I can't be friends with my superiors, it's not possible, I don't know how to deal with the hierarchy uh... and the... and the sort of connivance like that so uh... yes I'm getting used to it, I'm adapting to it, but I'm not uh... I'm not the... the first one to mess with my PU (University Professor) (#E14)

Do not question the seniors

there may be a chief who, at the beginning of my residency, uh... there was a little bit of pushing and shoving, but it wasn't anything personal in fact, it was on uh... uh... on... on strictly professional aspects either because he didn't like it too much when I questioned what they said or else uh. ... if I hadn't done something well rather than uh... telling me calmly, he might prefer to yell at me a little, but we never... well, it never got to the point where I found it uh... disproportionate or... or not justified. It was, that's all, reactions that were specific to the personality of the... of the individual, but which were... which were not excessive. (#E5)

Honesty with the leader

so here I think that it also passes by an... an honesty between the chief and uh... between the resident and the chief what also when there are situations a little like that what, when one asks us if one feels capable, it is true that sometimes one will have a tendency to also want to make some by saying yes, yes I am capable and then one is not necessarily, therefore it is necessary to have an honesty, (#E11)

To follow from to AZ

It's true that with this surgeon we get along well, so he wants to let me do it because there is a good relationship between the surgeon and the resident, because there is, because the surgeon has time, for example, there are few operations that day, so we'll take the time to let the resident do it too, for organizational constraints like that. ... it can also be because it's a patient that you recruited, so uh... it's a patient that you had on call, you accepted the patient, you took care of him, you did the brain MRI, you did the various uh. ... you did the different examinations, you saw the patient, you saw the family, it's a bit "your patient" even if you work under supervision so the surgeon tells you well here's this operation you're going to do, but it's not the majority of the cases and most of the time it's discovered like that during the operation almost that we're going to do it or just before uh. ... a few seconds before, but that's a pity because when you know it beforehand, the few times when someone was able to tell me "listen, you'll do this operation", etc., there is a little stress, but that's a pity because when you know it beforehand, the few times when you've been able to say to me: "Listen, you're going to do this intervention", etc., well, a little stress is created and you work on the intervention, you prepare it, you mentalize it in your head, etc. In the evening, you go to sleep and you think about this intervention and as a result, it's done in a way that's... well, it's completely... I find that the intervention is much more fluid because the steps are already in your head, it goes by itself, in fact,"... (#E11)

Difference between seniors

what I didn't expect at the beginning of the residency, is the difference between the two leaders, because in fact, uh, it's... all the learning is based on the... the seniors who are going to be with us and who are going to allow us or not to do certain gestures and to give us uh... theoretical comments

in relation to what we do in the OR or to... to keep their information preciously uh... until we are able to deserve it if I may say so uh... so I didn't expect that (#E12)

Solo with debriefing

there is an ordeal... a surgery that marked me at the beginning of my residency... which was a... difficult surgery that took place during the night shift... in fact... what happened was that my chief asked me if I felt capable of doing it, so objectively... I told him... yes, and I did it... I did it alone, it went well, and in the end, if it was a challenge at the beginning of my residency, it may have destabilized me a little at the beginning, but in the end, it was quite instructive and I found it very positive and in fact, my supervisor had a debriefing with me the next day (#E13)

Public danger

I never really understood how it happened in their heads uh... I never really understood uh... how they were sure that I was not a public danger uh... I... my conclusion is that uh... yes it's uh... immediate confidence, but I think from the moment they realized that if I didn't know how to do it, I would tell them I didn't know how. (#E14)

Companionship: trust and complicity

companionship afterwards is... it's like its name indicates, it's linked to... to a certain uh... trust and to a certain complicity between the... between the two companions so uh... it's sure that we don't have the same companionship uh... depending on the character in fact, quite simply (#E3)

Elective affinities

So yes, after everyone obviously has their own personality, um... but on the whole um... they let us do quite a bit um... and it also depends on the frequency with which we go with them, depending on our affinity for this or that um... type of intervention, we'll be led to be um. ... more often with such and such a leader who will know our level of progress and who will let us do it, so that's a parameter which is important and then uh... they let us do uh... things little by little so the transmission is pretty good. (#E5)

Different ways of seeing and supervising

companionship well, as I was saying earlier, there are different ways of seeing, uh... and ways of supervising, but I don't think there are good or bad, uh... bad ways of supervising, it's different things and each... each technique and each way of... to do has its advantages and disadvantages,

but uh... I think it's important to have a little uh... the big picture and to... to do the different things in order to have something that is a little bit, uh, complete at the end (#E9)

Honesty

so I think that it also requires an honesty between the chief and uh... between the resident and the chief when there are situations like that, when we are asked if we feel capable, we have to... it's true that sometimes we have a tendency to want to do things by saying yes, yes I'm capable and then we're not necessarily, so we have to have an honesty, (#E11)

Evolution of capacities

we feel as we go along that we are more at ease in the operating room, we have more... technical abilities and above all, uh... something that is important I think, is uh... knowing how to adapt to the... difficulties that we encounter little by little, but that's something that we have thanks to experience in fact, it's after having seen uh. ... problems arise and it's with the management of these problems that you learn to manage it yourself and uh... I find that this is really what shows the... the... the... empowerment and then the... uh... well we'll say the evolution of the uh... surgical capacities. (#E9)

A healthy relationship

There are... seniors who will leave a lot of uh... the old professor who have absolutely nothing left to do, well uh... they are rather relaxed uh... the chief surgeon who are a little stressed and who will leave us absolutely nothing at all even if uh... they have a lot of experience and that uh... they have always done it this way and they will always do it this way, and on the other hand, there are young people who have already passed the anxiety phase at the beginning of their assistantship and who will let us do things because they don't mind and because we get along. ... and that we get along well with them and that is also very important, it is the agreement uh... the expectation with the practitioner, with the surgeon uh... it is... we have the relationship of colleague to colleague, sometimes we were co-residents together so uh... it's things that... that are important, it's people that we esteem beyond work and that uh... bring a relationship to work that is often healthier uh... a little more uh... a little easier and uh... these people are more willing to let us do things. (#E1)

Companionship and complicity

companionship afterwards is... it's like its name indicates, it's still linked to... to a certain uh... trust and to a certain complicity between the... between the two companions so uh... it's sure that we don't have the same companionship uh... depending on the character in fact, quite simply. I

have a rather sociable character, I have never had any problems, uh... of discord with my bosses... It's true that there are bosses with whom I have much more affinity and with whom I go to work more often and that... and with whom I learn more than with others. It's true that compared to other colleagues of mine who have rather strong affinities or... or characters that are a little more... less easily sociable, it's true that, on the other hand, uh... they only go with certain people and they don't get along with certain chefs, so the companionship may... may not go as well with uh... certain chefs. (#E3)

Knowing each other well
So yes, after everyone obviously has their own personality, um... but on the whole um... they let us do quite a bit um... and then it also depends on the frequency with which we go with them, depending on our affinity for this or that um... type of intervention, we'll be led to be um. ... more often with such and such a leader who will know our level of progress and who will let us do it, so that's a parameter which... which is important and then uh... when they let us do uh... things little by little so the transmission yeah, it's pretty good. (#E5)

Nothing excessive
No, no, no. Oh no, no, no, there are no... well, in other cities we know that there are either madmen, or who put pressure on the residents all the time and who harass them in a more targeted way, but uh... I can't... no, I can't say that I've ever been uh... well, there was maybe one chief who, at the beginning of my residency, uh... well, he used to push me around a little bit, but it was nothing personal, in fact, it was on uh. ... uh... on... on strictly professional aspects, either because he didn't like it too much if I questioned what they said or if uh... if I hadn't done something well rather than uh... telling me calmly, he might have preferred to yell at me a little, but we never... well, it never got to the point where I found it uh... disproportionate or... or not justified. It was, well, reactions that were specific to the personality of the... of the individual, but which were... which were not excessive. (#E5)

Typology of residents
It's the resident who's going to do his residency, so basically it's the resident who wants to learn will look at the next day's operating schedule and he'll be interested, he'll read the patient's letters, he'll see what it is. Then there's another type of resident who, uh... well, shows up on the day, goes to the OR with a flourish, ah well hello... I've come to help... but in general it doesn't go well because the chiefs don't like it (#E13)

It takes time

... the more we work with them, the more they'll get to know us, uh... they'll show us how they want us to do things, and then they'll trust us and get us to do things. So uh... it's a bit of a shame that we change departments every six months so it's often at the end of the semester that we start to get to know them well... (#E15)

A good operating aid
to show that we... on the one hand we know how to help very well because if we help, we are a very good operating assistant for surgery... we know where he is in the surgery and therefore he can know that we understand the surgery, that we are capable of doing it (#E15)

Efficiency in services and in knowledge of the files
the fact of anticipating the surgery, i.e. discussing... with oneself, planning the surgery oneself, how I would do this... or discussing it even better with him the day before... to say here's what we do, we do this, we do that to see if we have understood everything and if we would have done the same (#E15)

And then also to show that we have... a good management in uh... notably in the service, of the occupation, of the perioperative, of the preoperative, we are uh... how to say... that we are devoted, that we have a self-sacrifice... well at work... that we are serious and stable, that's it. (#E15)

Availability
they say it, uh... it's... "if you have a problem, uh... if you have a problem with something, you call at any time, uh... well, there's no problem, we prefer that you call rather than keep something in your corner and uh... and do something stupid when it could have been easily managed... they all try to... to teach us the best they can in their own way and uh... and then if we have questions we can ask them without thinking that they are stupid questions and uh... and that behind... well... to look like a... excuse the expression, but "for an idiot" in front of the boss (#E17)

To gain the confidence...variable
How do you earn it... well, first of all, uh... by showing that you know how to do it from the start, by knowing the file well, uh... and uh... by being present both physically and... and by having discussed it with the surgeon, and also by drawing a line, there are seniors who, even if you're on the ball, will never let you do it, and some of them, even if you're not ready at all, they'll let you do it...(#E18)

A subjective point of view

There are some who will evaluate you a little bit on the feeling they have of you, but from a totally subjective point of view... (#E18)

A guy involved

the guy can be competent, but if uh... he doesn't see the patient in pre-op uh... he arrives late uh... in post-op he makes you papers uh... he fiddles with the papers... but technically he's good, well you don't want to leave him at all because uh... he's not invested whatsoever. On the other hand, a guy who is less gifted, from a technical point of view, and in terms of knowledge, but who is much more assiduous, much more invested, who prepares all the material, who sees all the points of view in the file, who does a perfect job in the post-op period on the follow-up, the thing, etc., you really want to let him do it. So it's both subjective, but at the same time a bit objective in the sense that it's the resident's involvement. (#E18)

A mutual interest

we are obliged to get along well with everyone and conversely the leaders are obliged to get along well with everyone, both for the sake of trust and not to damage the relationship because they also need little hands as much as we need to learn our job. (#E19)

The pleasure of leaving the hand

the leaders don't care about... what happens in the OR, so if they leave their hands, it's not because they don't want to work, it's uh... it's because they're happy to see that we're capable of doing it and that they can teach us something (#E19)

Don't hide your doubts

the important factors are uh... getting in touch with them if uh... if for example on the guards uh... are we square in the sense uh... apprehending the patient in a square way, we have a therapeutic proposal which is more or less coherent, that I think that it puts them in confidence uh. ... and I also think that one of the big elements is when we tell them for example... when we tell them "no, I'm not ready, I need more help". I think I'm part of a group that's a little bit, uh... I compare it to other residents, when I say "I don't know" it's because, uh... when I say "I know" it's really because I'm comfortable and sometimes I say I know how to do it, he comes to see and it's going well and that's it... (#E22)

2 left hands

It's a bit of a problem that I found in my residency, it's that uh... it's still a manual job and you say to yourself in fact uh... am I fit to do this so uh... you can say to yourself "yes I understand but I have two left hands you see" and... and that's complicated so sometimes I went to see uh. ... not

every day, but once or twice during the semester I went to see my bosses "what do you think, do you think that uh... I'm doing well, do you think that I have the right gestures, do you think that I have two left hands?" (#E23)

Getting on the learning curve

Afterwards, I find that the surgical residency is actually very complicated, it's to... is to situate yourself, especially in neurosurgery where you are one resident per class, so you have no reference, you can't compare yourself to your co-residents because you don't have any and if the chiefs don't talk to you too much, you don't know what they expect from you, so, um, that's always been a bit of a problem for me, it's been where I fit in the normal learning curve of a surgical resident who will be, um, a normal surgeon. I don't necessarily want to be the best, it's not in my ambitions, but uh... if you want, where I fit in the progression curve compared to a guy who will be uh... not bad what, and that actually is hyper difficult to do. (#E23)

Delegation of tasks

And I think that this, yeah, this, this allows you to gain the trust of a boss. I think the best way to lose it is when he asks you once, twice, three times for something that hasn't been done. I have an resident who, um, says to him, "Madame what's-his-name isn't doing very well, you could ask him for a scan," and I come back four hours later, and, um, the scan isn't ordered, and, well, that's a pain in the ass. You say to yourself "well then, I might as well do it myself". Which may be true, but... I wouldn't yell at him, but you see, you say it once, twice, three times, and then the fourth time you say to yourself, "Yes, I don't trust him, there's no point in asking him, he's not going to do it, so I'll do it". I think that the delegation of tasks, even if it is a little ungrateful at times, allows you to gain confidence. (#E23)

Finding your place

Nonchalance?

nevertheless, uh... with the same semester, or one or two semesters, there are residents who will operate more than others in the departments because they will be more appreciated, more integrated. So they can be more appreciated for several reasons, because they have a more pleasant behavior, they are less... there are some who are more... I don't know, who have a less adapted behavior, who are less hardworking, I'm saying anything, but it can be less hardworking, so arriving late to meetings, to staff meetings, to the operating room, it can be not knowing the patients, that is to say giving a sort of impression of... of nonchalance (#E11)

If there is a kind of nonchalance like that which can be... which can be badly perceived because it gives an obviously uh... negligent side of things when we are doing care, there are uh... people uh... it can be uh... it can be on the global behavior, that is to say people who answer a little, there are some. ...there are residents like that who are a little more "temperamental", who will respond, who will not be polite, I think that there is also something very important for the chiefs, it is uh... the... the relationship that we will have with the. ... the paramedical staff, so uh... that is to say that an resident who will uh... be available for the nurses, who will be of service to them, who will make sure that the service runs smoothly because uh... surgery is uh... not only the operating room, the operating room is a big part, it's probably the most important part, but it's... there's also the management of the patients before and after the operation, the relationship with the families, so the relationship also with the nurses, the health executive, the care assistants, the staff...(#E11)

The bad reputation

people talk about it, it's a small environment, the chief surgeons have been there for a long time, the nurses have been there for a long time, you're the resident, you're there for six months, so uh... people will quickly say well this resident is good, this one is not good, this resident is... she's a pain, this one is better, so there you go... so there is a sort of reputation very quickly, I think that it is created in three weeks... about three weeks - one month you quickly feel that there are... there are people who are categorized uh... who are put in boxes, he is good, but he is temperamental, he is good, he is nice, he is lazy and moreover he understands nothing... (#E11)

Services rendered

We're more likely to let go of someone who's going to be more present in the hospital, who's going to work more hours, uh... who's going to uh... move on, etc., we're more likely to let him do uh... surgeries than someone who's uh... who's always late, who's never doing what's needed... well... there's a kind of service rendered what. (#E12)

My little nest

Well, at the beginning I was a bit of an intruder and then, as time went by, I began to be part of the scenery and then later on, I found my little place, I found my little, I have my place in the elected program as well as in the urgent program, I'm starting to to have my little nest, but it was... but basically I was a bit too much, at least at the beginning (#E10)

With respect to the staff

we know that the surgeon is well above us and he is our... our master. On the other hand, what is difficult to situate oneself is within the paramedical team, uh... the nurses, because in fact we arrive as residents with uh... their background and their level, which they have, they have... once they are trained, so at the very beginning of the residency what is clearly difficult is to position oneself in relation to them because there is a... a sort of rivalry that is not always easy. ... a kind of rivalry that doesn't have to be, um... but there you go, so what's generally good is when the chief supports and affirms the position of the resident and says, well, no, the resident is a future surgeon who... he's the one who's going to do this time because it's his future job. (#E13)

To put oneself in the mold

I don't know if I'm old-fashioned or not, but uh... I remain deeply uh... convinced that hierarchical relationships remain fundamental in neurosurgery... especially in surgery uh... and that's what makes uh... I have the impression that I have heard this, and in [City N], the residents with whom things don't go well, not necessarily in neurosurgery, but in surgery, are residents who are not aware that there is an extremely strong hierarchy at the university hospital and that it is imperative to fit into the mould (#E13)

Prove your potential

First you have to prove that you're not dangerous, then you have to prove that you're useful, uh... and then you have to prove that you have surgical potential. (#E10)

Back pedaling

After uh... at the beginning they trusted me while keeping an eye on the... on the... we'll say on the results, but on the functioning, etc., and then afterwards it's a bit of a test too, if it... if we see that there are problems from the beginning, I think that there's uh... a backpedal and then we go back to the uh... more... less demanding level we'll say. (#E9)

Reputation

people talk about it, it's a small environment, the chief surgeons have been there for a long time, the nurses have been there for a long time, you're the resident, you're there for six months, so um... people will quickly say well this resident is good, that one is not good, this resident is... she's a pain, this one is better, so there you go... so there is a sort of reputation very quickly, I think that it is created in about three weeks - one month - you quickly feel that there are people who are categorized, uh... who are put in boxes, he is good, but he is temperamental, he is good, he is nice, he is lazy and on top of that he understands nothing... (#E11)

To be engulfed

At the beginning it was really uh... being there every morning, trying not to kill the patients and trying to cover all the blocks of all the chiefs because we... we were yelled at if they were alone without an resident. (#E10)

Service organization versus personal desires

We need a minimum of six residents to function properly, so each time it's a race to see which neurosurgery resident stays in neurosurgery, and which resident from other specialties comes in, and so given the state of things, our department head simply forbade us to leave the department. For example, I wanted to go to radiology, my co-resident was supposed to go to Tarbes to do an outpatient program in orthopedics and uh... it wasn't even discussed how it was going to be organized later on uh... in the continuation of our residency, if we couldn't do it next semester, we immediately said "well, next semester we need so many residents, so you're staying" and that's when we said that training was not at all, uh, not at all taken into account, not even by our chiefs (#E5)

The most suitable

there is necessarily a time when it's a job like any other, in all the other jobs there are interviews, I'm not shocked that if there are three people who want the job, it's not necessarily the best classified who takes it, it's among the three, the one who is the most adapted to the current team, to local needs, to local ways of doing things who must be taken (#E14)

Ungrateful tasks

let's say that I didn't expect us to be so uh... indispensable to the functioning of the service in the sense that I thought... ideally the residency, the expectation that we have, is that uh... we are there to be trained, to be taught things and it's true that in the end we are more uh... uh... how can I put it... elements that have to make the service function on tasks that are a little bit uh... repetitive, sometimes a little bit uh... thankless (#E5)

In the unknown

Even though we have an image of what it's like to be a clerk, it's hard to imagine what it's like to be a clerk and what we'll have to do before... I think we're going into the unknown even though we have an idea of what it's going to be like (#E9).

Claiming before proving oneself

there is a new generation of residents and the pivotal period, I think, was just after me, the two - three years that followed, so it's residents who have been here for two - three years, I've been at the university hospital for six years, but these new residents we are seeing now and they are residents who are a little bit different... so maybe... well, they are claiming more, but there are things... they are claiming before they prove themselves (#E13)

Resident status

Here we are, but uh... in fact I have the impression that people have not... have not understood that OK we are residents, we have a kind of status uh... of student, OK, but finally we are doctors because we have what we call the final certificate which is a certificate uh... at the end of the sixth year which gives us the title of doctor and in fact people they... they may think that we are students on the university benches and that we are not capable of doing much and this is diametrically opposed to the system for example in the United States where uh. ... the residents are... they have the title of doctor and in fact in the collective unconscious, doctor gives credibility and that's a little bit why I did my thesis too uh... before finishing my residency it's because uh... if I was alone on surgical procedures, I wanted to be able to present myself as a doctor and I tell them I'm doctor what's-his-name, I'm an resident, and the patients are reassured. (#E13)

Managing your schedule

you must not overload the theoretical part either, because uh... if you take away the on-call rests, uh... and the theoretical days, you still need to have days in the operating room to learn how to operate, because that's... how you really learn your job (#E4)

Mentors

I have my mentor
I have... I have my mentor I think that every resident uh... has a leader to whom he is closer than to the others without it being uh... deleterious to the others, but there are always affinities (#E10)

Heterogeneous mentors
what I didn't expect at the beginning of the residency, I think, was the difference within the chief... between the chiefs because actually uh... it's... all the learning is based on the... the seniors who are going to be with us and who are going to allow us or not to do certain gestures and to give us uh... theoretical comments in relation to what we do in the OR or to... to keep their information preciously uh... until we are able to deserve it if I may say so uh... so I didn't expect that (#E12)

Various personalities
I've always been much closer to the... well, the young practitioners, so the heads of clinic and uh... assistant heads of clinic, uh... who are often the people who supervise us the most, uh... on a daily basis, so with whom we operate the most, uh... and who make us do the most things, uh... and then, depending on the older practitioners, uh... it goes more or less well, depending on each person's affinities and personalities.(#E5)

To push to remorse
I've seen some very good things, some things I don't like, uh... probably people who are very good at what they do every time, but well... I've had services where uh... half of my co-residents have been very remorseful, uh. ... I've had others, uh... where it went very well, uh... they may become... well, future professors in their field, well, it's... it's very service and personality dependent, unfortunately. The personality of the leaders, not the residents. (#E12)

Follow your Mentor
For technical questions, for questions of operating schedule, uh... you just have to plan ahead. It's always the same, it's because I have a favorite chief and I'm his favorite resident, we know on Monday what we're going to operate on during the week, we talk about it, we go see the families, we make the post-op announcements, it allows me to be a little bit... but that's because I'm someone, but it wasn't the case at all at the beginning. At the beginning I was just coming into the OR, I didn't even know how it was done, which didn't make the learning process any better. (#E10)

Beginner's combo

The 3 interventions of the beginner

i.e., at the beginning, well, as I told you, these were the first three procedures that I was taught, uh... that I was taught, uh... at the beginning of the residency, so these procedures I systematically did alone, uh... I had mastered them, they had been shown to me, I had done them once with an old resident and then I had done them alone and then there you go, so quickly you... you know when these pathologies arrive in the hospital, you know that you are the one who is going to take care of them (#E11)

"Small" gestures

... after all, we're a center where they don't let the residents do much on their own, so I... what I can do on my own is really small gestures, putting in a chronic subdural external shunt, etc. (#E2)

Consultation with residents

subdural hematomas at the beginning we learn, but we don't manage them, but we quickly manage them from A to Z, that is to say that we are the ones who see them, uh... because we recruit them in emergency, so we are the ones who see them in emergency, we are the ones who program them, we are the ones who follow them. I think we can say that we are surgeons only when we take all these steps. That is to say, to see the patient before, to indicate him, to organize the operation, to do the operation and to follow him after the operation. If you don't do all that, you can't uh... say uh... I've... I've... I've already managed this pathology, it's not possible. You have to have done all that. So uh... to know that, you have to have the consultations or something to... we'll say recruit the patients, that is to say the emergencies, you have to operate and then you have to follow up afterwards, so often you have to have a consultation so it goes through having a consultation and we have a consultation of residents, we often manage.... well we manage the spinal fractures uh... to operate or corset and the subdurals. (#E3)

The simplest gestures

we are empowered for the tasks that are uh... essential to the functioning of the service, but uh... we are not left to do anything either. From the second semester onwards, I think we start to be autonomous for the simplest procedures, ventricular shunts, etc., chronic subdural hematomas and then little by little we do more and more procedures, and when we are able to do a procedure on our own we will be able to do the operative report and the coding so that's... that's from the second semester onwards I'd say and then gradually. (#E5)

Yeah, so there are operations that I was allowed to do quickly, which were, uh, operations, uh, for example, subdural hematomas, shunts or other operations that are the first surgeries to be done on the ward, uh, which are fairly quick, uh, which are not very complicated technically. (#E8)

Beginner's overview
I just had to learn the first procedures, so the procedures that you learn in neurosurgery in the first semester, in order to acquire an autonomy that is very fast, that is to say that in one month you have to know how to do these gestures, we'll say these first three interventions, so it's the installation first of all of the... of an intracranial pressure sensor, so that's probably the first thing we're taught to do, then the installation of an external ventricular shunt, the second thing we're taught in chronological order, and finally the third thing we're taught. in chronological order and then finally the last one is the evacuation of a...the evacuation of a chronic subdural hematoma uh...so these three procedures were shown to me uh...so initially mostly by...old residents who were with me (#E11)

Day 2
Ventricular drain and subdural hematoma, they like the... the chiefs to teach this to the... to the... to the younger ones more than they teach them because in addition... because it's gestures that do more simply, EVD uh... (External Ventricular Drainage) it's uh.... the ABC of neurosurgery... it's the most urgent and uh... the most... that is to say that we learn... I learned that on the second day (#E3)

Total disinterestedness
there are a few procedures that, uh, are of the order of total and absolute disinterest for almost everyone, which are mainly chronic subdural hematomas and external ventricular shunts, and which are procedures that are shown once or twice and then, uh, you're on your own, big guy, uh...(#E1)

We try as best we can to do something that resembles what we have learned or at least what we have seen once and that's where I think that my biggest uh... my biggest uh... my biggest despair we'll say is that this training was sometimes a little uh... light on surgical gestures. (#E1)

Accelerated training
Yes, yes, but it's in training, uh... it's... it's accelerated, that is to say, we watch once, the second time we do uh... with the chief holding our hand, the third time he watches us and the fourth time we are definitely alone. (#E13)

At the beginning...
that is to say, at the beginning, well, as I told you, it was the first three interventions that I was taught, uh... that I was taught, uh... at the beginning of the residency, so these interventions I systematically did alone, uh... I had mastered them, they had been shown to me, I had done them once with an old resident and then I had done them alone and then there you go, so quickly you... you know when these pathologies arrive in the hospital, you know that you are the one who is going to take care of them (#E11)

Putting it all into perspective
It's quite important and then uh... afterwards there are also the first times when it happens in... on call at night when we do a procedure that's not very complicated, but the first times when we have to do it alone after the... we operate with an on-call system in [City N], so with the chief who is not on the premises but who is at home, uh... there is still a certain amount of... of stress uh... that's not negligible and then little by little we... with the habit and with the... uh... training, we manage to relativize a little bit all that, but yes in any case it's something which is very... which is very progressive. (#E9)

Basic tricks
Well, there are quite a few surgical... I mean, there are quite a few surgical procedures that are, uh, more, uh, maybe not simpler, but maybe faster, but in any case, they're done earlier in residents. ... things like that, and then there's all the rest, in fact, of the surgery which, um... on which we can perform two or three, um... how should I say it... stages of the surgery which are performed much later during the residency (#E15)

On your own
yes, a little bit, uh... we do... quite quickly I started to do external ventricular shunts on my own in... in the ICU. I had been taught the procedure a few times before, and now I'm starting to do subdural hematomas in the O.R., on my own, with several times the chiefs or older residents showed me first, and then I did it in front of them, and now I'm going to start doing it on my own, on my own, on my own (#E17)

Hygiene and common sense
there are two... two interventions in which we participate from the beginning all the time since they are undoubtedly interventions that are uh... at any time, that are quite quick and that do not require a great deal of expertise, it is the ventricular drains and the chronic subdural hematomas... this type of intervention, uh... easy, not particularly technical, that could be performed by uh... anyone provided that they know the rules of hygiene and common sense. common sense. These two we let us do from the start and they don't require extraordinary technical know-how (#E19)
The basic interventions

The person in charge will inevitably be there in the operating room, she won't leave us full responsibility to delegate it to someone else so uh... I would say that we can always tell afterwards how we did it, we can always take a video on the microscope, detail what we did or uh. ... yes, I don't know, tell uh... in detail uh... what happened, but training and teaching surgical practice from resident to resident, that's not done very much outside of extremely uh... extremely basic procedures. (#E19)

Heterogeneities of services

Heterogeneous training

I'm convinced that the level is not homogeneous, that there are cities where the training is better, and cities where it is worse. Of course, the individual plays a big role, the investment you make in your work is... I think it's largely responsible for that, but it's... there are probably also... I think that half of it is the individual, the other half is... it's the teaching, and after that, we have to identify the factors that make the teaching good...(#E11)

Habits of services

so probably it's also related to the service, there is also this factor which is very important I think, there are services where they will let you do more things and other services not, in particular in [City P] where there are several services, there are nine neurosurgery services, so everyone has their own habits and therefore lets more or less the... the residents do. (#E11)

Difficult start

I thought I would have access to theoretical training, more easily, more structured, more square, and even oral training, easier to access, but in the end, it's up to the residents to go and get the information themselves. there is no neurosurgery resident book, I think it is much less uh... square than in other specialties where there are very clear recommendations, in neurosurgery it is not the case, it varies a lot from one city to another, from one operator to another so we don't have already... on the theoretical level we don't have much to base ourselves on and even more so as a young resident because we don't know how to use the tools of research, etc., well medical articles, etc., (#E12)

No European exam?

I think it would be good to have much more theoretical training, uh... I think it would be good to have exams with a program and exams and program that is to say, uh... I think it would be good to have exams with a program and exams and a program, that is to say, uh... not each one of us will do our own courses and we will try to answer the questions afterwards, but an official program for everyone, so with guide lines, and I think it would be good to be evaluated because, in my opinion, the level of residents is extremely heterogeneous in France, and I think that the level of French residents, compared to other countries, is much lower, because in all the other countries, uh. ... there is the European examination which is compulsory... well which is compulsory and which you have to pass to be a neurosurgeon which we don't have at all in France...(#E12)

Stimulating policy

I'm in [City M] right now, this is the policy that is in effect, I think it's great because each resident has the right to say well I'm pediatrics, I'm vascular, I'm the base of the skull, I'm oncology, and they all go to train elsewhere and all go to inter CHU and come back with specific skills that the others don't have, and that makes for a very homogeneous group and a very good general level because in fact everyone is stimulated by someone (#E12)

different activities in different cities

We don't use it too much, uh... uh... it's not used too much, and then after this kind of model that I had seen of what intervention we are supposed to do in such and such a semester, it's not always, uh... reliable in the end, there are... well, there are things that we'll do earlier, things that we'll do later... it's a matter of... of time... well, or activity depending on the city, so uh... well... (#E4)

Comparison

I recently did... well, I'm currently doing an residency... what we call an inter CHU residency, so... an exchange in [City T] in another department... after that, I think it's quite heterogeneous depending on the... depending on the department. I found that in my current residency, in another university hospital, there is less autonomy and you are less abandoned from the beginning of your residency. (#E9)

Stratifying the training

I think it would be good to have a... a personalized follow-up of the resident with uh... I think pedagogical objectives to have each year. For example, um... the first year... well, it's always a bit special, we'll say the first two years, knowing how to do everything that's urgent, third year, starting to know how to do um... well, it would be good to have a sort of program with objectives um... (#E12)

Heterogeneous level

I think it would be good to have much more theoretical training, uh... I think it would be good to have exams with a program and exams and program, that is, uh... I think it would be good to have exams with a program and exams and a program, that is to say, uh... not each one of us will do our own courses and we will try to answer the questions afterwards, but an official program for everyone, so with guide lines, and I think it would be good to be evaluated because, in my opinion, the level of residents is extremely heterogeneous in France, and I think that the level of French residents, compared to other countries, is much lower, because in all the other countries, uh. ... there is the European exam which is compulsory... well which is compulsory and which you have to pass to be a neurosurgeon which we don't have at all in France... (#E12)

At the goodwill of what's-his-name

on a practical level, uh... well, to have objectives set for each semester that are met, so that we have a little more, uh... an overall view... well, so that we know a little more where we are and what... so that we have a progression to make and not that it's at the whim of, uh... what's-his-name, uh... so that we know a little more... I've got six months to learn how to do this, learn how to do that, learn how to do that, and for the bosses to stick to it and let us try. (#E7)

We should discuss beforehand

I think that already, uh... we should systematically, which is not done in any case, uh... locally here, eh, after it is perhaps done in uh... other neurosurgery units in France, eh, uh... but I think that we should systematically discuss all the files, well all the patients who go to the operating room and say uh... which approach uh... the head how uh... well why we operate it what... well I mean, what uh... what is the interest and then uh... of the indications and uh... of the uh... of the techniques, of the approach, uh... really analyze, I think that the analysis uh... of the preoperative imaging in fact does uh... it does a good part of the job in fact uh... I think we don't realize it, but uh... I think it's... well, when we have the imaging in mind, well, I think we're already less confused during the surgery and uh... and we can do without modern tools like neuronavigation, etc. I think that's the first point. (#E6)

Late learning

Those left behind
Ah, no, no, the proof... there are some residents who left the department and were never given a chance... (#E10)
there are some chiefs who were in the department at the time and did not trust him either for the shifts or for the... the operating room so he finished his residency without having operated. (#E10)

A heavy atmosphere
I found that to learn at the beginning it's easier to learn in the... in the... we'll say in a relaxed atmosphere than in an atmosphere that is uh... that is heavy. That's the term, an atmosphere that is heavy because the pathologies are more serious, because the procedure is higher risk and because, well, um, people are less tolerant of error, obviously (#E11)

Late start
I was expecting uh... how to say... maybe to operate, to practice more or at least more... more quickly and uh... to feel... then I realize that uh... we'll show you a lot a lot before doing... it depends on the cities, but in any case in [City L] uh... we start operating very late uh... in the residency or even rather in the clinic or the assistantship what. (#E15)

Closing
I was in the mode where we were going to practice a part of the surgery and then another and another part, etc., until we could be a little bit, uh, autonomous in all the surgery, but in fact, uh, in the beginning, we were almost only made to practice closures, even almost during the entire residency, so, uh, only closures, many closures, closures... (#E15)

The heart of surgery
the heart of surgery itself, the most interesting, well whether it's for tumors, tumor cases or uh... for uh... for the skull or for the spine the placement of screws, etc., that's much more... well much rarer. (#E15)

I was watching

when I was a student, I went on residency several times, and it was a center that had a habit of quickly letting go of the hand in the operating room and of I didn't know that it was a dependent center... and in fact in Angers it's not at all the same... the same teaching philosophy so... I was a little surprised to see that I had... that I didn't operate at all at the beginning of my residency. I was looking at it (#E20)

The specialty of my dreams

Lucidity

The more I gain in... in modesty, in humility as I progress, the more I realize that uh... here we are, we are still quite helpless, we rarely save people and uh... and at the beginning I absolutely wanted to be someone's surgeon, now I take advantage of the time I have as an resident... by saying I am an resident... I am young...(#E10)

My passion

...really the medicine I do finally uh... bored me a little bit compared to neurosurgery and then neurosurgery in itself fascinates me as much as... even more than I thought, that's it (#E15)

My vocation

I never felt like a surgeon...the first time I set foot in an operating room...I said to myself "ah this is made for me". (#E16)

My criteria of choice

there were few... few specialties that fit these two prerequisites... manual labor and investment. (#E19)

My science fiction movie

It happened... it happened pretty early because even uh... if it's not necessarily you doing, the first time I saw awake surgery, I was like fuck but I'm in a sci-fi movie it's unbelievable what, so on my first semester it happened pretty quickly, yeah. Then uh... given that we have big responsibilities uh... that... on... on the really concrete stuff, I would say in the department of... problems... of putting intracranial pressure catheters uh... on your second week we send you to drill skulls alone in the ICU uh... pretty quickly you say to yourself "yes, okay, I'm doing something special"... (#E21)

Operative pedagogy

The hard task of teaching

I find it very difficult to succeed in teaching without... without sounding pretentious so uh... I admit that... most of the time I try to avoid teaching because I don't know how to do it at all. (#E1)

Harmony in surgery...

Harmony in surgery is a sweet illusion...there is no clear consensus on this kind of thing so even if there was, I'm not sure it wouldn't be considered because they would consider that...that it's not necessarily what they have learned so uh...it's not necessarily what they consider as...as the truth whatsoever. (#E1)

Patience and past bullshit

I think that the best training I received was, uh, with people who were patient and, above all, who were very aware of the path they had taken before. of your career, remember that we did all this nonsense. (#E13)

The Grail

I have the impression that it's uh... it's terribly difficult for a lot of chiefs uh... you've got the chief uh... who tells you to do it all by yourself, he shows you and if you don't succeed, he takes the instruments away. You have the chief who does everything, who explains to you what he is doing, but you don't do anything so uh... from the outside you have the impression that you have understood, but the day you have the instruments in your hands, well it doesn't work and there are very few... chefs who are able to describe everything they do while leaving the instruments in your hands and I think that's the... the Grail (#E14)

The ideal moment

it's true that with this surgeon we get along well, so he wants to let me do it because there's a good relationship between the surgeon and the resident, because there's time, because the surgeon has time, for example there are few operations that day, so we'll take the time to let the resident do it too, because of organizational constraints like that... that (#E11)

it's of course the leaders who judge, I imagine, according to, uh... competence and what they believe we are capable of doing and assuming, uh... but it's at the whim of the leader too. Even for the same chief, uh... one day he will let us do an operation and uh... I don't know, we go back to the same chief for the same operation ten days later and for X or Y reason, he won't let us do it.
We don't do the same man the same gestures for the same surgery and in fact there are little things, types of tricks that you can learn from other chefs for even very simple surgeries and so I find it and so I think it's really good that there's always a chief who lets you have a hand, doesn't get dressed, etc. Uh... and that afterwards there is a companionship, so I think that this is also a key to practical companionship, it's the surgeon's patience, well that's... you can't make a man out of him, (#E11)

It is difficult to be patient
it's true that having a patient surgeon, even if I say that I think I'd be the most impatient of surgeons, so I don't want to say that it's something easy, it's not a criticism that I'm making, it's a criticism that I'm making globally about the human being, it's... it's very difficult. (#E11)

Retention of competence
The surgeons are not necessarily willing to teach us directly, which is quite understandable, but I think that there is a... There are leaders who will not explain to us what they are doing, for example, and as long as we don't show that we have worked on the subject, etc., or that we have been there for six months, we will not be able to explain what we are doing, or that we've been here for six months and we're starting to take an interest, it's important not to give us too much information so that we don't progress too quickly, but I've seen that quite a lot, eh? (#E12)

The one who sulks
so I told him no, I can't do it alone. He didn't really like it and in the end he did his patient like a grown man who didn't need me, he wasn't very... very educational, he could have shown me once and told me that afterwards you'll do it on your own, it didn't happen like that, (#E14)
There is not only one way to teach well for me there is not necessarily a good or bad way to... to learn, but I know that... well in the surgeons we work with we know that we are used to and that we know that they will not all function the same. (#E9)

Take over the hand
if you operate a little too slowly or if you don't do the right thing or you don't do exactly what he had in mind, etc., he will take your hand back. This is something we don't like very much, we often say it between us... well, he took my hand back, uh... too bad, it had started well, etc., he took my hand back because this, because that, (#E11)

Pan on the fingers
There is one of my bosses... my department head... who at the very beginning of my residency was... very demanding with me... and there I felt that it was indeed a little stereotyped behaviors, but which were reproduced because in fact he would tell me... "well, me of my age". ... "well, in my time with my boss, um... if I didn't do things like that, I'd get a needle holder on my fingers; well, if you do anything at all, I warn you, you won't be treated any better treated," (#E13)

Alone with the carotid
he says to me, well, listen, you start, I'm coming. During my third week of residency, the world was already falling on my head, I was no longer with my friends, I had left my city. ... well, it was all nonsense and he told me to put the carotid artery on the side and move forward... I'm not ready to forget that one... so I told him no, I can't do it by myself. He didn't really like it and in the end he did his patient like a grown man who didn't need me, he wasn't very... very educational, he could have shown me once and told me that afterwards you'll do it on your own, it didn't happen like that, (#E14)

Technical progress
So I started with a first semester of orthopedic surgery where, uh... very quickly, it's true, we let you... we let you have a hand in the operating room, uh... we check that you know how to do the basic movements, so the first knots, the uh... whether it's the simple separate stitches, the overlays... So it was a gradual learning process, I would say, over a month or so, where, uh, all the movements were really supervised, we checked the... the quality of the knots and I think I had a... well I was quite happy if you like with the companionship in the operating room the first semester because it was in a specialty that was not mine (#E11)

Natural progression
gestures that I found complicated at the beginning of the residency that I did under supervision, so I wasn't stressed or in an uncomfortable situation because I always had my chief with me, and that was something that reassured me and that, I think, made it go well. really, I really felt that at the beginning of the residency, a kind of... uh... it was something quite natural, that is to say that as soon as I felt that my limit had been reached, I felt it because I said to myself, I don't know what to do, so finally the chief said to me, it's okay, listen, I'll take your place, I'll take over, and then little by little it was done (#E11)

Chief present in the operating room

there is also a somewhat theoretical side that we often forget, anatomical, theoretical and then finally that uh... well that in the operating room... I think it's really good that the chief is there uh... I think it's very important even uh... even if there is trust and even if the resident can do it alone. I've never been offended because a chief was there in the room, I've always been happy, even for easy interventions, even if he doesn't necessarily get dressed, even if he's next door, I like it because I feel... well, it reassures me, (#E11)

Take over the hand
you operate a little too slowly or if you don't make the right gesture or you don't do exactly what he had in mind, etc., he will take your hand back. This is something we don't like very much, we often say it between us... well, he took my hand back, uh... too bad, it had started well, etc., he took my hand back because this, because that, (#E11)

Dad on standby
You did like a surgeon, you took charge of a patient from A to Z like you will do when you will be... when you will be chief so you feel like a little chief while knowing, you know you say to yourself if I have a problem I can call "dad" what, you know, it's a kind of feeling a little bit of... it takes away a stress in fact. (#E11)

Skipping the basic steps
In fact, the older you get in the residency, the less you have to, uh, have done all the preliminary steps of this same surgery before you can do it on your own, but at the beginning of the residency, yes, it's... it's... in general, our training is done like that, yeah. (#E4)

Practical transmission
Most of the time, so the transmission, it's that uh... they transmit more easily on emergencies than on a programmed, but roughly speaking, so uh... either at the beginning they explain what they do and then they'll start by making us do the simplest steps uh... here by watching what we do, by giving us vague advice and then once it's acquired uh... these steps, they will let us do them alone or even without being there and then let us do other steps in their presence by helping us or advising us as needed, so uh... here is the transmission for the practical uh... technique, it is quite... it is quite good in our service in the sense that we are made to do it progressively, we are explained to us, we are taken back uh... and we... here we are let go little by little until uh... we know how to do it correctly uh...(#E5)

Questioning the best method

Uh... I don't think I have enough mastery of the surgical procedure to be able to uh... talk about learning it uh... I have... I have my young co-resident who is always asking me I don't know uh... I don't know if constantly explaining what they're doing makes you learn faster, I'm not so sure, explanations as you go along I'm not sure you print more than when you watch uh... I don't know if you have to do it very early or if you have to take your time. I think that, uh, more and more residents are doing it early and I don't have the impression that... I don't know, frankly I don't know. I have... I did all my procedures late, I looked at them a lot and I don't think that it was wrong for me, and at the same time, I hear all the residents today saying that if I don't operate, I'm not going. I find that incredible because uh... I think it's important to look and so I'm a little confused about... about what's the best way to learn, I don't know. (#E10)

Starting in another specialty
So I started with a first semester in orthopedic surgery where, uh... very quickly, it's true, we let you... we let you have a hand in the operating room, uh... we check that you know how to do the basic gestures, so the first knots, the uh... whether it's the simple separate stitches, the overjections... So it was a gradual learning process, I would say, over a month or so, where, uh, all the movements were really supervised, we checked the... the quality of the knots and I think I had a... well I was quite happy if you like with the first semester in the operating room because it was in a specialty that was not mine... there was a very relaxed atmosphere, it was orthopedic surgery, so it was very... we operated with music, the chiefs were very patient, uh... it was... there was an atmosphere. ... they were relatively young surgeons too, uh... a fairly young team and so there was a sort of atmosphere like that which was uh... almost friendly and so it's true that it... it gives you confidence and it facilitates the learning process in my opinion at the very beginning, especially when you're very stressed about uh... anything. (#E11)

Dexterity is learned
Well, to be a good surgeon, dexterity is important, to be able to use your hands, but it's something that can be learned, like everything else, because no one, uh... well, train at home to manipulate already with very fine gestures, it's true that it's not something you do, so it's something that is learned in the operating room and that, well, it's something that comes, I think, quite naturally and it's not necessarily the most important thing, it's not necessarily what makes the. ... the... the "greatest" surgeons in terms of results, there are surgeons who can be uh... very, very meticulous, but who will take a very, very long time, and so of course that will lead to other problems (#E11)

The incredible patience
It's difficult for a teacher surgeon to... uh... it's patience, you have to explain, say no, it's not that procedure, look, I'll show you, I'll take the instruments, I'll do the procedure without... without doing it, I'll simulate it, I'll give you the instruments back, you do it. That's for sure... it's an

operating time that is... that increases by 1.5 or even doubles, so uh... well if you do it like that, but uh... it's for sure that the resident comes out, he says it's incredible (#E11)

The right sequence
I learned best when I was left to do it, so uh... after having observed several times, I think it's important to let go of the hand, because even if afterwards you spend several surgeries being an observer again, already the fact of having done it just once, well, you don't look at the same things anymore, you don't look at the same gestures, you're more interested in the same things, because you try to transpose the gesture of the other person to your own hand, so it's still very different (#E12)

Let me do it
I liked the chefs who let me do it, but really even if it meant debriefing before each time, we talked about it, but who weren't stuck on me, correcting me, jumping on me because I was doing something they hadn't imagined or according to their habits, uh, that they'd had for decades, that's it. (#E13)

The chief and the spreaders
Uh... the department head was like that apparently in the past, but now he decides he's too lazy to operate, which I understand because he's at the end of his career, so I didn't benefit directly from it, but once by chance he got lost in the blocks, he saw that I was all alone uh. ... on a herniated disc and that I was having a hard time uh... he got dressed, he held the retractors for me, so great the head of the department who holds the retractors on uh... the herniated disc and he did exactly what I just said and frankly it was a huge help and I didn't need much more (#E14)

At the helm... with a boss on the side
the way... the way I learned the most was uh... when uh... I was the one doing it and the boss was next to me and said uh... yes, it's good what you're doing, go on, do it like that or no, be careful, look at her, uh. ... I don't know, there's a vein, uh... either you have to preserve it or uh... you see, it's useless, so clot it and that way you won't make it bleed, uh... it's in the hands of a chef. I think that's really how you learn best (#E6)

The steps

our leaders are very difficult to have us at the beginning when we are clumsy and we do anything, we have to be uh... very careful about what we do uh... then there is a period when they are happy that we help them and then things are going well and then at the difficult period they have to teach us things, they have to be, they have to have confidence in us and that actually that... that's done in stages. (#E10)

The small downside

the small downside is that we're not sufficiently, uh... it's not organized enough, if you will, the companionship in the operating room is a bit, uh... when I can, when I want, that's it, I would say, for the chief. The delegation of the surgical gesture is a bit when I want, when I can, it's not organized, it's a pity, that's the big weak point, I think, in France. (#E11)

Do not forget the theory

there's also a theoretical side that we often forget, anatomical, theoretical and then finally that uh... well that in the operating room... I think it's really good that the chief is there uh... I think it's very important even if uh... even if there's a trust and even if the resident can do it alone. I've never been offended because a chief was there in the room, I've always been happy, even for easy interventions, even if he doesn't necessarily get dressed, even if he's next door, I like it because I feel... well, it reassures me, (#E11)

Cutting the threads

after I have the impression that we... maybe we let me think more about what we are doing, the planning, the installation, trying to... to know what the next step is going to be, and gradually they let me do some... parts of the surgery, but very gradually, and then it really depends on which surgeon I'm working with. There are some who... who... for whom we are always just the operating assistant to water the field, to cut the wires (#E2)

The perfect stitches

On the other hand, uh... for example... well, it really depends, I think, on the... senior surgeons, but... there are some, uh... who, uh... who still have trouble closing the skin, uh... whether it's me or older residents, uh... because everything has to be absolutely perfect and, uh... we can make a few stitches, but they'll be removed in the end if they're not perfect... so... (#E2)

The best method

in the end, well, there is a good balance between the surgeries that we do alone, uh... certain chiefs with whom we have more, uh... complicity, I would like to say, or who will make us do things, uh... quickly... I think that, like everywhere, there are seniors with whom, uh... I prefer to, uh... to go to the operating room, uh... I operate a lot with the clinical leaders because uh... they let me be the operator alone and they are there to uh... uh... to brief me live so I find that it's... it's the best surgical training, it's to do all that, but with someone who comments a little live uh... what we do. (#E4)

Live debriefing
The... the... the older seniors will gladly let me do uh... blocks on my own, but they won't necessarily be there. So that's good too, it can teach, but what's missing is uh... I find uh... well then the coaching or the live debriefing of what we do. (#E4)
What is missing at the beginning gestures that I liked... that I would have liked to do and that I didn't do, it's actually at the very beginning of the residency, it's basic gestures but learning to hold a Kerison, learning to... to drill, to learn how to make a hole, well, we only learn that by seeing and we learn late to do it ourselves (#E1)

2 types of surgeons
there are two types of surgeons. There are those who let things go and who go back to the smallest detail every three seconds and who in fact end up taking over and in fact they can't stand to see someone do it badly so they interrupt us every two seconds and there it's unbearable and there's the surgeon who lets things go, even lets things go a little bit so that... so that the resident realizes by himself that it doesn't work and I think that's a better learning technique. (#E12)

DU of pedagogy
I'm saying that, I'm taking a university diploma in medical pedagogy, but it's theoretical pedagogy, not practical surgical pedagogy. It could be interesting to have a... a surgical pedagogy, that could be something interesting (#E11)

By feel
Uh... I've never had any... surgeons who tell me: "Here you go, you do the first step systematically, uh... because you have to know how to do that, or you do it afterwards... then you have to know how to do that, you have to know how to do that..." No, it was really: "Listen to this patient, you... it was a bit of a gut feeling. (#13)

The ideal help

I think the best way to learn is to extrapolate a little from what I experienced in the medical services, but uh... it's... uh... with someone who is physically present and uh... and who pushes you to find the answer and the solution and in surgery, that's how I see it and... well the best surgeons I've ever met, in my opinion, were people who were able to stand next to you, not to touch the instruments, to let you do it, but to say ah well no, you should do it like this, I'd rather do it uh... I'd do it like this and to know how to describe these gestures (#E14)

Create the reasoning

Yeah so I would say that, then really create the reasoning. Then it would be to... Listen, if as a chief, as a trainer, as an academic, you say, here's this resident, I'm going to let him operate, listen, well, here's, uh... you're going to operate, you're going to operate on this patient, so the resident should already know that. Okay, I'm going to operate on this patient, great, I'm happy, uh... very good, uh... so you're going to operate on this patient, you'll have to operate on such and such a date, etc., how would you do it... so that he can uh... that you can as an resident already develop your reasoning (#E10)

Our topics of interest

The second element that intervenes in priority is uh... training, so we'll try to... to go on the interventions uh... which interest us more or which are rather in uh... the type of activity which we would seek to develop or to make later uh... here it is... it is the criterion which makes that very often thus we make several days in advance which intervention we are going to make and thus obviously it is much more interesting to uh. ... to look at the files beforehand, to look at the images, to try to see what type of technique, of approach we... we are likely to use, if it is an approach with which we are not familiar, possibly to review it beforehand so it is obviously uh... it is more interesting to... to anticipate which block we're going to be on (#E5)

A real companionship

I think that the second point, um, is, um, it would be, for example, um, to... bah to have a real companionship in the uh... in the operating room by saying uh... bah there today uh... it's you who does and I'm next to you and uh... and how you would do it... well really prepare a work of preparation indeed upstream and then uh... and then really carry out uh... the D day uh... the thing and then when we are in difficulty uh... to have somebody who advises us, etc., and not uh... hand over at the first difficulty and then when you're really in too much trouble, obviously uh... have the boss next door who is there and who allows uh... to overcome that difficulty. (#E6)

A visionary

but I think that it could be quite good to...in fact that there be a real companionship uh...between a chief and an resident and uh...that it be for example uh...for six months uh...the same binomial I want to say and that uh... I don't know, one day or two days to go and see in another center how they do exactly the same type of things they do, whether in France or abroad, and that we bring back certain ideas or things... certain ideas or certain things here, because it's us... well I think it's generally like that in the surgical community, it's... anyway we are the best, we know how to do things better... .and we don't question ourselves... but uh... well, that's not what surgery is about... so... I think that this could be quite original and uh... I think that the guy who sets it up will be a visionary. (#E6)

Don't neglect the practice

I think that uh... we lack a bit of theory. In Lille we do a lot of practice, that's very good, but we learn, uh, we learn a little bit on the job... well, yeah, anyway, and I think that it lacks a little bit of... for us in any case, it would be good if we had... we have meetings... for example once a week we have meetings for spinal surgery and all that uh... but it would be good... well if we had a little more theory, I think... the other staffs, I think for example of all the neuro-oncology meetings, all that, uh... the files go by and we say well radiotherapy, chemotherapy, but no one's going to... it's just a fact that we're doing this, it's just the protocol that makes us do this, but the goal is not to be educational, the goal is just to show the file and choose the next step in the management, so I wouldn't include this in the educational process, whereas the other staff we do, um, for the spine, is. (#E8)

Staying present at the resident's side

there are some who think they can do this by talking a little and letting the resident do the surgery without being there, but unfortunately this is not the best way, even in certain centers where residents operate alone, but they still learn from contact with patients and on the job, so to speak, whereas the best thing is of course that the senior is present and that he... that he is... that he tells them what to do in the surgery...(#E15)

On a practical level, it's to be there from the beginning to the end of the surgery and to guide all the procedures. (#E15)

All or nothing

In fact, with us it's a bit all or nothing, it's either we let you do it, or you help and you don't do more, there's not really a version of it, uh... you start with four hands with the chief who lets you do more and more and... there are others who do that, but on the whole, uh... three quarters of the surgeons I still hold the suction to do very little and the retractors uh... in any case I'm more efficient, I anticipate things more, well here's uh... I

know what's going on, but uh... but with us it's more like learning to "you saw, you saw, you saw" and then all of a sudden uh... "You're going to do it and then you don't call me if you have a problem" (#E16)

I hold the vacuum cleaner, I'm not much use and then uh... and then one day "well go ahead, it's your turn". (#E16)

Not easy to let go of the hand
it's people who have trouble letting go and when they operate they want to operate themselves so they realize that and they know that they have to have the residents operate and so when they think that the resident is ready... they let go of his hand because they know that if they are there, they... they don't do it. (#E16)

Technical strategy
a first part that I... I identify because I find it lacking is a theoretical preparation of the gesture during the training of... not so much on the indications which we learn quite quickly even if we don't necessarily have a lot of courses on the subject, but rather on the... the choice of why I'm going to approach this thing from such and such a place, which structures to pay attention to, etc., in other words, everything that is the first pathway and how we decide to approach a tumour, an aneurysm, etc. (#E16)

Learning and restitution
That is to say, I have a chief who will make me do the skin, muscle and bone, but for example, I never asked myself how they were going to incise the temporal muscle on the skull and... and then the other chief next door who, when I'm with him, only gives me the aspirate to hold, but then he'll... I suddenly realize that I've seen it two hundred and fifty times, but in fact I don't know which way I should cut, uh... it allows me to... well, it's... it's a bit of a learning process, restitution (#E16)

Technical debriefing
we may not have enough... comments on the intervention itself, i.e., we are in the process of performing an operation X or Y and... it's quite exceptional for someone to tell you "ah well, there's such and such a structure that you have to pay attention to", etc, So it's something that ends up going in, but which would go in more easily if they had shown you the thing once and for all and told you "well, this is the nerve" or "this is the ureter" or "this is the... I don't know". (#E16)

Binomial

In [City A], the residents follow a chief and only do uh... well, they follow the same chief all the time for two months, so they don't do any other operations uh... except for on-call or other concerns, for example, there aren't enough residents and someone absolutely has to come to an OR, but really for two months they follow a particular chief and I think that uh... it's another way of understanding things and maybe that too uh... well... I haven't tried it personally, but uh... maybe that's also much more formative since it... it induces uh... how to say uh... a transmission what from his senior towards us since for two months he works only with us and uh... he knows where we are in our training and he can let us do this or let us do that and uh... and according to the affinity he can create, he can uh... there I think that the transmission is not the same as when we go to anyone at any time. (#E17)

Binomial 2

by imagining a surgeon who does two days of surgery a week and who does more or less the same surgery or the same surgical theme, and he has an resident who follows him for three months, yeah, clearly that can happen. He can really train him. (#E18)

Empirical...

How is the surgical gesture transmitted? uh... well, for me, to summarize, it's in an empirical way, that's it. (#E18)

The best technique

The best technique is a particular chief - I'm not going to name him - it's a chief who simply uh... puts himself next to you uh... we have a microscope where we have uh... on one side the operating surgeon and on the other side the assistant and this chief systematically lets me uh... to be in the surgeon's place, to stay next to him, to look at him and to guide me on my movements, i.e. either advice or instructions on how to place the instrument, how to place a retractor, how to place a vacuum cleaner, etc. We continue to work without ever intervening. I think that's really the... with him it's clearly the moment when I learned the most because he's in the background, well he's constantly present next to and in the background of the surgery, he doesn't take the instruments, he doesn't put himself in the place of the operator, and he tells me when he sees that there's a need to... when he sees that I need help... then he looks at it and that's uh... yeah it's really great, that's when uh... I learned the most with uh... someone next to me who is able to refrain from taking the hand and redoing the gestures and manages to be uh... at the same time precise enough on what needs to be done without being uh... without being annoying of micro precisions and uh... of parasites what. (#E19)

Discreet support

once you... you're going to be a little more uh... autonomous, once they're going to let you do a little more, what I like is when the leader is uh... he's there, but he's a little forgotten. He's there, he watches, and then at times he'll tell you, um... he'll correct your actions a little, he'll... he'll redirect you or if you want to ask him questions, you can ask questions, but he still lets you, um... do a lot of things. (#E21)

You don't learn by watching videos
He was able to stand back even in times of difficulty uh...working with a big risk with the patient and it's clearly in those moments uh...where I learned the most. It's by being confronted with difficulties, it's clear uh... if we're faced with a breach and we're taken back to manage it, well we never learn how to manage it and if we're taught surgery by showing us, well we don't learn as well as by doing it... I think you must have heard it dozens of times, but uh... we don't learn a manual profession by watching videos or by watching uh... the colleague uh... operate. You learn by taking both the risks and... (#E19)

Encoding by repetition
I'm learning to prepare him on a theoretical level uh... in the OR we try to see him often in close proximity, that is to say uh... if we see a block uh... and if there is the same uh... the same type of intervention two days later, it's good to have him seen a few days closer together, I found that it made it easier to encode things than if we saw him once every six months. (#E20)

The patience of the senior
Afterwards, I have my department head with whom I also get along very well, um... the advantage is that he lets me operate on the shifts alone, basically he's not in any hurry at all on the shifts, um... because there are some heads who basically want to go home, um. ... and uh... if you operate at three in the morning and it lasts an extra hour because it's the resident, well that doesn't bother him, he'll stay in the department in the middle of the night, but uh... he won't pressure you at all, he'll let you do it (#E22)

Make the gesture
I think that uh... the best way to learn a procedure... clearly you have to do it! (#E22)

Confrontation with practice / The archetype of the gesture
Afterwards, I think what is good at the beginning is to have a small theoretical base, but which is very... at the beginning, which is not necessarily... I think not necessarily very advanced, eh, the big essential points, the things that determine the archetype of the gesture... afterwards, I think that it

is necessary to see it once... and then I think that once you've seen it once or twice, you have to be confronted with the practice because it's by doing the practice that you realize that there are a lot of questions that... that you didn't ask yourself before, that you didn't even understand why it was important, etc, so I think you really have to uh... you really have to be confronted with the practice uh... (#E22)

The logical progression of the gesture

which is sometimes not easy for the leaders, um... it's um... when you do it, you have to let the... the resident sort it out a little bit more, um... in the sense that, um... well, at the beginning, it's... there are a lot of questions that you ask yourself, which are not necessarily, um... relevant, but which you ask because you haven't yet done it. ... the logical progression of the gesture, uh... but the fact of asking them, it... it helps you understand, uh... for the gesture and for other gestures afterwards, uh... that's it, I think, that's the best thing, it's a little bit of theory and then practice, we let you sort it out a little bit (#E22)

The patience of the teacher

when it's just young residents learning to make gestures and it's true that at the very beginning you have to... you have to force yourself by saying uh... I don't see it like that, but uh... but in the end what he's doing isn't dangerous, it's... it's not what seems to me to be the most efficient, but you have to give him a little bit of time to... to each one of them to get their hands on it and... and to see what he does too (#E22)

The pressure of the senior

ninety percent of what I see uh... I would feel like doing it almost alone and in fact strangely the fact that I have... I have planned the thing and I want to do it, the fact that there is a chef in the room sometimes makes me operate less well because in the end uh... you see, if he's just in the room, if he doesn't get dressed, if he's just watching from a distance, it's a bit disturbing because he's really watching what you're doing, you're not necessarily doing it the way he wants, even though you'd have the same result, you're not using the same instrument as he is at the same time, and as a result he's always there making little remarks and I find that it's actually a bit... it's a bit of a hindrance to you in your planning. (#E23)

I am a manual

let's say that when I don't do it, after a while I find it hard to follow, that is to say that I am very manual and if you want if I look at it after half an hour... I'll think of something else because I can't, well I never watch TV uh... so there are some who say that they learn a lot by uh... the fact of seeing the operation uh... well if they manage to do it, good for them, it's not my case because if I stay at the back of the room watching, I think that after twenty minutes I'll think about something else or I'll be on my phone or uh. ... really, after that it's a question of mentality, I'm not the

kind of person who just sits around doing nothing, I'm more into the manual than watching others... I think that the best way to learn is to see what you do, to do it yourself, to understand why you didn't succeed and then to do it again afterwards, and to see the leader again. (#E23)

The right sequence
a little bit in fact and I think that the best way to learn is to have done, to have failed and to try to understand by seeing the leader again why you failed. And when you've missed a screw three times, well, the fourth time it's in and after you've understood how it goes in and then the following ones are good, eh? (#E23)

The best way to learn
I think the best way to learn is to make a mistake. So putting a screw 2 mm next to the pedicle, if it's in there, it has no consequences for the patient, I'm not talking about... I'm not talking about cutting an artery or killing the patient, but little things like that I think that's how you learn, yeah. (#E23)

...unique to each person
If there are people who can stay focused for three hours at the back of the room, watching every move, and those who will tell you "oh no, I have to see how he does it, how he takes his instrument, etc.". I think it's... it's specific to... à... his way of thinking about residents too (#E23)

Finding time

Spatial and temporal variability

That is to say, during periods when there are many emergencies, when everyone is a little overworked or during the summer vacations, we know that this training is less present, and when there are enough people, and when there are enough seniors, and when the service is well organized and runs smoothly, we know that we have a good number of staff. we know that we have a little more training in this area, we are a little more experienced in this area, we know that we have a little more training in this area, we know that we have a little more training in this area, we know that we have a little more training in this area. on that side, we are a little more taken care of, but that's a little variable and it depends a little... well, it varies according to the time and according to the local capacities...(#E9)

Theoretical training included ?

... you have to motivate yourself and it requires ... to work a little bit outside of our working time because normally we have ... we have training days, uh... that are included in our... in our weekly time, we'll say, but that we don't necessarily have the same amount of time depending on the activity of the service. (#E9)

Operating logbook

Quali vs quanti

It depends on... because I have my mentor here, so um... who does longer skulls, so I don't know if the... the number of operations is a good... a good indicator of the time spent in the OR because um... it's one thing to do four herniated discs or replacements of Parkinson's batteries, and it's another thing to do a neuroma that lasts ten hours, it's just one operation. (#E10)

A punitive thing

I think that the surgical logbook would be interesting if there were regular checkpoints, if we had a chief at a given moment who would tell us to come, we'll open your surgical logbook and we'll see where you're at in terms of surgical procedure. Yes, I think it would be interesting, but if it's just a kind of punitive thing at the end, where you get an unpleasant reflection, uh... the day you become a surgeon... (#1/13)

A stupid punishment

all my former residents experienced it like that, as a kind of completely stupid punishment where the day we are going to pass our diploma of specialized study, the jury will look at our operating book and will look down on us and say... you have operated a lot, you have not operated a lot, you don't know how to operate... (#E13)

No pressure

it's true that the... the chiefs don't really look at what's on it, I think it will be important at the end of the residency for the presentation so that uh... I can justify that I have a certain volume of operating activity uh... that's really uh... very autonomous on that, but uh... I fill it out, but it's true that no one pressures me every six months to make sure that it's up-to-date. (#E5)

In the blur

already in my model, there was supposed to be a regular assessment of the operating logbook, to see how things were progressing... we can also imagine interviews every six months to see a little bit... what progress I've made, how we feel, what our objective is for the next semester, what shortcomings my chiefs have seen, what gaps they have identified in terms of... and all this we don't have at all, we're completely in the dark in fact about uh... what we've learned and about uh... the evaluation that the chiefs have of... of our acquisitions in fact and so that I think, there you have it, an evaluation on the theoretical level which should be organized nationally with exams uh... (#E5)

Interest of the notebook
I think it's a very good thing because apart from the final diploma evaluation, I note down quite a few details that already allow me on the one hand to... to have a notion, well, on what percentage of activity I have, whether it's in the spine, in the skull, etc., or others, what percentage of activity I am, uh... operator or first aid or second aid, etc., so it's really, uh... a good tool. (#E15)

In fact, in total, I'm at six and a half semesters, I'm at 1021 surgeries, including 501 cranial surgeries. 501 cranial surgeries, 423 spinal surgeries and then the rest, well, other surgeries, and I think I counted the number of... In fact, I don't have the number of first surgeons in the operating room, first assistants, second assistants, and overall I made a little graph and it's true that you can see little by little over the course of the semesters that the percentage of second assistants decreases, first assistants as well, and finally the percentage of first operators increases. So it's also indicative that there's a gradual, um... autonomy. (#E15)

Lack of logbook
there is no operating logbook and there is no follow-up, um... it's perhaps a somewhat disjointed learning process in the sense that we'll let you do something, um... maybe not, um... we'll let you do more complex things before simpler things, or we'll realize that you're in your fourth year of residency and um. ... you know how to do a lot of skulls on your own, but most of the spine you are still an idiot because uh... it's always the thing that they let you do or not let you do, so that means that it's... an apprenticeship that at the beginning can be a bit of a patchwork quilt...(#E16)

Element of learning
To be able to say to oneself at the end of the residency that one has done uh... so many surgeries in... of such and such a micro-specialty... well, it also allows you to remember a little bit... each case and... yeah, it's also part of the learning process to say to yourself... well, there you go, I've seen ten... let's go look at where the difficulties were and... and there you go. The old people keep a logbook, um, also for the purpose of... for research purposes, to have a list of... of all... having a register of all the patients we operated on from a particular uh... sub-specialty (#E19)

Taking stock from a distance
that's really the point: to be able to say to yourself, um... I would say that... even personally to say to yourself "well, I've done um... fifty herniated discs, um..." well that means that you've been confronted with most of the problems, you know how to manage most of the situations and that, um... there you go, you know that you've acquired a certain degree of autonomy. (#E19)

It could be useful

I think it would be useful if there were a real consideration of what's inside, i.e., if we say "well, such and such an resident has done very little of this type of operation, well that means that on the program, uh... he'll do a little more, etc." That I think would be the way to go. That, I think, would be the most intelligent way to use it, to see where you operate more, or less, after I... I know that in Amiens we don't operate like that at all (#E22)

Heterogeneity

Heterogeneity between centers and men
For example, in the operating room, um... depending on, um... I don't know, I've worked with, um... with about thirty different surgeons since I've been in four different hospitals, um... there's not at all the same access to information, to procedures, um... to... well, not everyone is a teacher in the same way... (#E12)

Those who do not help
Uh... not especially. After all, there are some leaders who will never let anything be done because that's how they are, that's their way of doing things, they've already been trained that way, so they don't want to give too much too quickly because they haven't been given too much too quickly, or because some people are too stressed, so it's their patient, it's their responsibility, so they can't let go. to let go, it's really uh... very dependent person (#E12)

No regulation
The resident chooses his city and his department under the impression that he understands how things work and then he will find himself either in a department like mine, which is very permissive and where, uh, you are very quickly very autonomous, or in another department, uh, I don't have an example in mind, but I would say Paris, well, you don't do anything and you don't touch anything until the end of your fifth, sixth or seventh semester, and that, in fact, there is no regulation (#E14)

The seniors will judge
Uh... I think it's also very important, uh... well, the first times when you operate with uh... with a senior, where you have to prove yourself a bit, it's certain that uh... between two different residents, uh... you don't necessarily do the same things at the same time in your training, depending on uh... what the... what the seniors think you're capable of doing. (#E9)

Elective affinities
I've always been much closer to the... well, to the young practitioners, so the heads of clinic and uh... assistant heads of clinic... who are often the people who supervise us the most on a daily basis, so we operate with the most... and so they make us do the most things... and then, depending on the older practitioners... it goes more or less well depending on the affinities of each person and the personalities of each one (#E9)

Wishful thinking

CadLab

ideally, I think that we should organize uh... I don't know, for example, one of the residents uh... each time with... different approaches, different themes. I mean, I think it would be... it would be good in the training of residents to have that on a regular basis. (#E15)

Ideal abstract

as I was saying... more practical teaching, uh... in the operating room as I explained, with the presence of the senior from beginning to end, and letting the resident do, uh... certain gestures, one part, then another, then the whole surgery, etc. Maybe also more training sessions on cadavers in the lab, why not also simulations and perhaps more theoretical discussions, but on surgery the day before with the senior (#E15)

I think that courses, um... theoretical courses, um... organized, more frequent training days than we have at present, once a year, um... workshops... dissection workshops, um... and, um... perhaps imposing a little bit, um... in all the centers, um... end of residency interviews, um... surgical objectives to be acquired in such and such a semester, I think that this could, um... (#E20)

Valuation of the senior

I think that, uh... that, uh... that the chiefs should be much more responsible for the training of the residents, and that they should be valued in one way or another. (#E18)

The U of University Hospital

the chiefs should all become aware that when they work in a hospital, in a university hospital, they have to uh... they have to train us, so they should all understand that uh... well, the residents should be allowed to do it, that it should be more uh. ... more like I told you at the beginning, uh... that certain chiefs are... who are committed to training the others, but that everyone, uh... everyone should know how to let go of their hands on this or that problem or this or that part of a surgery. That's a mentality that's not... yet acquired. (#E19)

Clear training objectives validated by an evaluator

to see a logbook, uh, structured with clear objectives and with a referent for these objectives ... Rather than, uh, rather than it being a bit unspoken and something vague, uh, throughout the training. Someone to refer to who can say, "Well, you're at this point of... show me what you can do on a standard case". (#E19)

The importance of anatomy

I must admit that I would like there to be a little more space for, uh... well, for... for dissections with the heads in the lab and, uh... and then some courses really in... of pure theory, notably from the anatomy where I find that uh... before they were certainly better trained... well trained differently in any case from what we are, and with several co-residents including other specialties uh... of my generation, we realize that we are much less good in anatomy than our chiefs and that they were at the beginning of their residency and in fact there are many moments when it blocks you and so this is something, I think, that could be important uh... it could be important yeah. (#E21)

Having more time

basically, we still have a lot of shifts and it's still... I think it's still... I think we'd learn more... we'd be more effective if we had a little less, to rest, to take a little more time to read articles, etc., and we're not a big hub, uh... skulls, rare things, we see very little of that, but for the time being, uh... basically, uh... training for a general neurosurgeon, I think it's the best thing, uh... in relation to that (#E22)

The importance of to be on-call

Seniority on demand

we have an on-call telephone, we sleep uh... on the spot and our chiefs go home or not depending on the confidence they have in the resident who is on call or depending on their conviction. There are chiefs who never go home, regardless of the resident, there are chiefs who always go home where they will only do shifts with old residents and there are chiefs who go home more or less depending on how the shift is going. (#E10)

Emergency vs. scheduled

but I think it's generally the same everywhere, uh... where you, uh... emergencies are often operated on by the resident, that is to say, scheduled surgery, when you go to see... you have a pathology, you go to see a surgeon, he says I'm going to operate on you, it's often he who operates on you, even though sometimes he can delegate certain tasks to the resident, sometimes everything, but it's rare. On the other hand, when the patient comes in through the emergency circuit, i.e. through the emergency department of the... of the hospital center, of the periphery or of your hospital center and who is then transferred to the service that you recruited as I was saying earlier, when you recruit a patient, very often you are the one who operates on him; whether it is to operate on him during the night or to operate on him a little later and so uh... so uh... it's true that we are... we are often brought to operate on emergencies...(#E11)

Important training

for "basic" urgent pathologies, very often it's the resident who does it and then, depending on his level, uh... if it's... if it's... if he's too young to uh... to go all the way, then we let him do a little bit at the beginning and then we take over from him, etc., so I've found that for emergency training it's good because personally I feel I'll be able to manage the surgical emergencies that will come my way the day I become chief, (#E11)

The freedom of the guards

So it's a kind of freedom, but not too much... I like to be framed, but relatively free all the same. You have to assume that when the patient is operated on, you tell the chief, you don't act like a cowboy and you don't operate, but what's good is that during the shifts you have autonomy over your thinking, that is to say that the chief is not going to be there to make our work easier and tell us to do this, that and that for the patient. It's always like that, we get the call, we see the patient and in fact we're the ones who think and then we contact the chief and suggest something, so in that sense, the on-call duty is very good and very formative. (#E13)

Relieve the chief

on the practical training uh... it's a bit oriented in the sense that we are taught very quickly to do uh... which will allow us to relieve the chief a little bit uh... during the shifts, etc., but uh... on the interventions maybe programmed uh... where the chief doesn't necessarily need us to help him or do it for him uh... there we are much less autonomous, much less uh... involved, I would say (#E5)

Nychthemeral variations

I think during the day we were not as concerned about my skill level as we are at night at six in the morning uh... we may be more likely to be dropped to do an external ventricular shunt or something that is a little uh... that is accessible to a two - third semester resident uh... there you go but if it's six in the morning, maybe the chief will be less inclined to come than if it was in the middle of the day, so that's true that empowerment can vary a little bit depending on those parameters. (#E5)

A milestone

there was a surgery that marked me at the beginning of my residency, which was a difficult surgery that took place during the night shift, in fact, what happened was that my chief asked me if I felt capable of doing it, so objectively, I told him yes, and I did it. I did it alone, it went well, and in the end, if it was a challenge at the beginning of my residency, it may have destabilized me a little at the beginning, but in the end, it was quite rich in lessons and I found it very positive and in fact, my chief had a debriefing with me the next day (#E13)

We are not cowboys

we have to start from the principle that when the patient is operated on, in all cases, we warn the chief, we don't act like cowboys and we don't operate, but what's good is that during the shifts we have autonomy in our thinking, that is to say that the chief is not going to be there to make our work easier and tell us to do this, that and that for the patient. ... it's always like that, we get the call, we see the patient and in fact we are the ones who think and then we contact the chief and suggest something, so in that sense, the on-call duty is very good and very formative. (#E13)

Elective

Basically, these are patients who are doing well, they have seen them in consultation, there is a personal commitment, a guarantee of results, etc., but they still let us do, etc., especially for tumors, so, etc., it's more prudent, I would say, but, etc., it's still done through transmission. (#E5)

it's when we're on call and we do... and we operate on call that the chiefs let us do the most, especially because... basically there was no relationship of trust between the patient and the chief that... was established given that it's an emergency, whereas leaving us a patient... in the cold block, so that was scheduled, etc.., they are a little more reticent because if there is a problem... well, they are the ones who are, uh... well, the patients know them and trust them. (#E7)

Quite free

again, it depends on the chiefs, there are chiefs who really let you do things and then there are chiefs who let you do few things, so uh... it's a bit chief dependent, but uh... overall, the emergency room is really uh... except for uh... things that are really uh... well technically difficult uh... such as clipping aneurysms or things that are not, uh, within the capacity of the resident, uh... overall, I'd like to say that we're quite, uh... how... we can be quite free with regard to, uh... with regard to emergency pathologies, uh... (#E6)

Major interest

In fact, you can't... like... you can't pretend to be... after being... in neurosurgery and on a senior on-call schedule without having done a lot of on-call duty as a junior beforehand. (#E15)

Minor interest (old resident)

Well, in fact, the emergency room is not very complicated, in the end there are two, three, four things to know... to know and then uh... the rest is not urgent and so it will wait uh... or it will move on to something else so in fact uh... the gestures are not complicated either because they are simple basic gestures that concern everyone uh... so uh... yeah... no... typically I did forty-eight hours of guard duty this weekend, I spent fifteen hours in the operating room yesterday, I don't think I learned anything... (#E16)

Empowerment factor

There's always a chief on call and an resident on call, uh... at first we call them all the time, then we call them less and less, then we call them, uh... when we've pushed the patient into the OR or almost, uh... in itself, there are no particular difficulties on this subject, um... there is an old chief, um... who grumbles and says, "don't call me after nine o'clock", but in practice, if you call him, there is no problem (#E16)

Guard versus Room

it's really formative I find uh... it's where you're the most uh... the most autonomous uh... it's really where you're the most autonomous uh... and uh... likewise all the little things you have to do uh... often they're done on the guards and uh... I find that it's really where it's the most formative. There and also in the operating room of course. Where it's the least formative is when you're in hospital for a week managing patients, it's not really what you're going to do later, it's... it'll be the resident's job later...(#E17)

Unselected patient
On call and in the emergency room, that's where you learn because in fact it's not a patient who's been selected, seen in consultation, where there's no real contact with the chief, so he's much easier to let the resident do it. (#E18)

You are afraid but...
Yes, you're afraid, but, uh, but generally they let you off the hook on surgeries that you know and, uh, and I've always had that, that is, I've always had a chief who was present, that is, if I say to him, "I'll need your help, I don't know how to do this, can you stay in the OR," I've never had, uh, any... people who didn't come, you know. people who didn't come. (#E18)

Emergencies not interesting
emergencies are... uh... it's really stupid stuff, it's kind of surgical cases uh... it's all stupid surgical cases, neurosurgery emergencies are uh... there's... either it's broken, you have to uh... you have to... repair, the repair is not very complicated, it's... it's a matter of unravelling (#E19)

The field of residents
I don't know... if you say it out loud it... it sounds strange to me, it sounds a bit like... we don't care about the patients on duty when it's not that at all, but... but in practice it's true that we... the residents operate more on duty than... than on scheduled patients. (#E21)

Gaining experience
we have a lot of autonomy in the sense that we are the... whether it's on the... the indication, the management, the referral, we are basically on the front line, you have to be very autonomous uh... you decide if you want to transfer people, if uh... but yeah you have a lot of autonomy uh... for the on-call and then you are confronted with a lot of... well, it rings all the time, so you have a lot of... uh... you get an experience that is uh... that is hyper important because basically uh... on a shift I don't know, you're going to deal with maybe uh... thirty files, you've maybe operated on two - three patients so on each shift it's uh... all the more so if you do it every three days. (#E22)

I really remember having had... I think at the beginning of my second semester that I had basically done more than one semester of neurosurgery, if I compare it to other specialties, I had already been in a lot of situations, I had already been in a lot of cases and when I compared it to other specialties where they did a lot less... (#E22)

Post-call rest

Friday night

Uh... but yeah I think that... doing his consult on a Friday... doing a chief's consultation on a Friday night when you're off duty, when you're tired, when you can't take it anymore, but you'll never say no. (#E10)

Small remark...

Then uh... the... safety rest has a lot of uh... there have been stories, there have been quite a few things in the media, things like that where I think there has been a kind of awareness and then there has been a kind of fear also of the heads of department, of administrative... and I think there has been a pressure for this rest to be respected, especially in our specialty uh. ... and so uh... overall then... what I feel at the moment is that safety rest is tolerated, that is to say that no one will say anything to you, they will make a small remark saying uh... you are fragile, you are tired, go and rest uh... there you go, they will make a small remark, but no one will blame you. (#E11)

you have the department's big learning policy, which is not always perfect, and so they end up with residents whom they do not consider competent, but whom they do not want to take the time to train, and so, um, I take my on-call rest on an um, on a day when there is a complicated block for a chief, and he sees me leave and says to me, "You're not coming to the block with me? I tell him no, I'm going off duty, but there will be X, there will be I don't know who, I'll say any name of the resident... ah no, but that's annoying, who cares about on-call duty...(#E14)

Not to get carried away

Then there's always the problem of on-call rest, I think that's really one of the things that comes up most often... I'm the first to say that you have to take your on-call rest because you don't work in good conditions, but it's always the same, you don't get carried away and uh. ... and advocate the residents' union card by saying I warn you, if I don't have my on-call rest, I'm going to take it to the unions because I'm convinced that when you do it like that, it doesn't go well, (#E13)

Organizational plasticity

I'm in favour of safety breaks, of course, but... on condition that, um... it's necessary... well, it's not necessary to take it systematically either, and that the other residents find themselves, um... not numerous enough because we're not there (#E15)

The same speech
It was always the same speech, the safety rest, it's that uh... you have the right to it, there's no problem if you take it, but uh... it's a shame to... to miss out on some of the uh... on the safety rest what. That's a bit of it. And then there are always old... old fogies who say "in my time we had one shift out of two, uh... we were there every day, it was no problem, that's how we learned life" and then you see that these guys and then you see that these guys now leave at 4:00 or 3:00 p.m., uh... who aren't around much, and they don't want to work anymore, so it puts them in contrast. (#E18)

A year lost
if you're in a department with four or five residents, which was the case during my entire residency, well there are four or five of us on the on-call schedule, so on-call every four or five days, that means uh... that means uh. ... more than once a week, that means more than uh... fifty days a year, that means that over five years, it's uh... one year of training that is lost in on-call rest so we do... we don't do it for a lot of reasons. (#E19)

Everything would be worse
we're understaffed and if we're not there, there's... there's no one in the OR and there's no one in the OR on... on interesting things and so uh... everything's a shame so uh... we lose experience, the chiefs lose help and things uh. ... in the service are not done... it's a bit of a circle uh... it's a bit of a vicious circle what, it's... we could, we could leave, but everything would be less good and for us and for the service. (#E19)

On the advantage of post-call rest
we said to ourselves "fuck, today I shouldn't be here too much, I haven't slept too much, I'm just going to go to the OR to have a look" well all those little half-days of scratching around, going to the OR, looking at the patients... going to the OR, looking at things, saying to yourself, "Oh, I'm supposed to be off, I'm not going to bother with the service, I'm not going to make the rounds, I want to do what he's complaining about, I'm not supposed to be here anyway, so, uh..." but in fact that's where you learn, I think. (#E23)

I think that on-call rest should be a bit specific to each resident, if you feel like going home, you go home, if you don't feel like going home, if there's a block that interests you, well, you go and have a look, but uh... but I think we're doing something stupid if we go with these new laws and say "wait a minute, you were on duty yesterday, at quarter past nine I don't want to see you at the hospital anymore"... well, you see, if there's a very interesting operating room that you want to see, well... I don't know why you wouldn't go and I think that's part of the training (#E23)

Companionship between residents

Generation

Uh... I don't get along at all with the resident who is two years older than me, who is above me, but not at all, it's really hell, uh... so it doesn't really make life any easier, and then with the two younger ones below me, uh... it's very, very nice, they call me Mom (#E10).

A relaxed atmosphere

I didn't expect to find this, I expected to find a very rigorous world of work and uh... and the atmosphere wasn't... it wasn't that at first, it was something much more uh... light and uh... centered around the... we have a good time together, uh... that's it. It surprised me a lot at first, I had a lot of trouble at first (#E14)

Symbiosis

as we don't have the same interests, finally I transmit to him... things that... that I know in my fields of interest, but he also transmits to me things in fields where I'm less competent, so uh... as we have a slightly smaller age gap uh... important, the transmission is bilateral, it's not uh... it's not unilateral, vertical from me to him because I'm older what. (#E5)

Elective affinities

I try to let him do it, not to take his hand away, to assume that he's already seen the intervention, so I'm not going to show him up, I really have to let him do it and uh... if I see that he's doing something uh... that doesn't seem right or... or that could be done better, I point it out to him, but I try to let him do as much as possible by giving him explanations and uh... and then in the transmission we have another co-resident who is not in the specialty, who is an ophthalmologist, whose name is Y, but who uh. ... is in neurosurgery and who, well, finally has a lot of facility in neurosurgery and perhaps we transmit more to him with my co-resident Romain than we transmit to the resident uh... of the specialty ... we have more affinity with him, we also have the impression that he understands better in discussions, even if it's only on the indications, he understands more, he's quicker-witted, so uh... there you go, maybe that... that we transmit in a somewhat unequal way to... between the two co-residents. I try not to make a difference, but it's true that the... uh... relational aspects mean that uh... I go faster if I have an resident who seems simple or that I feel comfortable (#E5)

Starting with residents

At the beginning, I spent a lot of time in the operating room in the emergency room, which allowed me to... I saw them essentially first of all by residents, so I had the aspect... the visual side that was already done... and then when I was able to take the first neurosurgery shifts after about three weeks of residency, I think, so uh. ... three weeks of residency in the second semester uh... I was able to uh... to do these first procedures for these operations from the first shift, moreover I was able to do the three procedures with a chief who had uh... who was with me and who had uh... who had guided me through the procedure, who had dressed with me. (#E11)

It shouldn't be mine

I can't say that it's unpleasant, but uh... in fact I consider that uh... it's not my role as an resident to uh... to do that. There are heads of clinics who should normally be trained, uh... well, train the residents, it should be the heads of clinics who train the residents and not the residents who train the residents, (#E6)

Chaperone the co-residents

well, when I arrived, my older co-residents were with me for this type of operation, the classic on-call operations that I was talking about before, and even for more complicated operations... well, for surgeries, for example... I start with one of my co-residents who is younger and then we... we... and then the chief comes to see how things are going, etc. (#E8)

To compensate for the senior staff

he can't take shifts for the moment and we pointed out that he wasn't making much progress and that it was going to be complicated in the semesters to come if we didn't teach him more, if he didn't progress and so we...well I expected the chiefs to take the lead and uh. ...well, I expected the chiefs to take the lead and, uh, train him a little better, I don't know what to do, if it was on a theoretical level... well, in any case, they were interested in knowing what was wrong and... and, uh... proposing solutions and, in fact, the only, uh... the solution that was proposed was precisely that... that my co-resident Romain, who is in his third year and I am in my fourth year, take care of teaching him emergency procedures and how to put in an intracranial pressure sensor, an external ventricular shunt, and that we have the impression that... that... well, we let him drift a bit, we ask the older residents to teach him the basic gestures so as not to disturb a chief during an on-call, but I don't have the impression that we have measured the seriousness of his shortcomings and the lack of transmission to this co-resident. (#E5)

The basis of our profession

Afterwards, um... our job is still based on transmission from the older to the younger and independently of each person's functions, so um... it's a bit implicit, we're not there... well, we don't teach the... younger residents in an organized way, but we teach them to do the basic things that we ourselves were taught, um... that I myself was taught by the older residents.

Not my job
well, for example, the first semester resident who is told, uh, well, he's going to put an EVD in the ICU and he's never done, uh, who calls for help from one of his co-residents, it seems obvious to me that I'm going to go and help him, I'm going to show him how to do it, but in fact, uh, it's not our job. (#E6)

We stick together
It's going well because we're here to stick together, we're here to uh... we're all in the same boat so uh... if... if there's someone who's having a little more difficulty, we're here to, to help him, to pull him up, to explain it to him. (#E1)

Practico-practical
I'll leave teaching to other colleagues who are more oriented towards that and who have more of an academic bent than I do, but uh... from there to explain... to train on gestures, on teaching things that are a little more uh... practical-practical, it's not a problem at all and I'm fine with that, but in purely theoretical teaching, it's not... it's not my field. (#E1)

Mom
Uh... I don't get along at all with the resident who is two years older than me, who is above me. But not at all, really it's hell uh... so it doesn't really make life easier and then with the two younger ones below me uh... it's very very nice, they call me mom. (#E10)

Transmission
On the other hand, between me and the resident who... who is younger than me, who is a year younger than me, they let me teach him to do... to do his first EVD so I was... I was with him for his first EVD. For the brain biopsies as well, because it's... it's a procedure that the residents do on their own, ah... ah yes, that on the other hand... that on the other hand, it's... it's Z, the resident who's older than I am, who taught me how to do the... the biopsies, yeah, for that procedure, and so I... I taught D, who came a year after me (#E2)

Whatsapp
there's someone who says, well, this is a problem, um, someone has to go and operate on something or go and help something and... Well, in our team of residents, it's not a problem. We say it on Whatsapp and there's always someone who'll say well listen, I'm free, it's a bit boring, but go ahead, I'll do it and there you go. (#E3)

Supervision
I went to training sites that I already knew, where I had already been, so I knew the... the functioning of the service, the interventions, etc, so yes, it happened quite quickly that I was supervising young residents in the departments for medical problems, but also in the operating room where there was one of the senior staff who told me that he had to go to the operating room. one of the seniors who says to me, well listen, you go to the OR with your, uh, with your co-resident, you show him, I'm the same, I'm there if, uh, I'm there if there's a problem, if you have any questions, but you both do it (#E9)

Qualitative problem
by dint of having, uh... the not-so-old resident teaching the new resident, well, in the end there were things that... well, that weren't quite done right, uh... so... uh... the chiefs kind of took over certain things, and then, uh... this year essentially, a little bit last year. (#E16)

Primordial companionship
the little things we do in the ICU, the external ventricular shunts, these little things, the first times, it was my co-residents who taught me and afterwards, it's already happened that a senior, a young assistant could teach me, uh... also taught me the gestures in his own way, but uh... all the little gestures uh... that are done by the residents, we'll say that it's uh... the older, senior residents, who teach them to the young residents. So actually also uh... they taught me things.' (#E17)

We don't do a lot of bonding
again, that's what I'm telling you, anyway it's necessarily healthy and we necessarily get along well because the workload is so high that we can't do anything but uh... get along well, but... yeah... I wouldn't know what to... what to tell you either, I don't have much to tell you, it's going well, but we're building uh... not a lot of bonds because we're working a lot actually. (#E18)

Basic gestures

in fact, the gestures that I mentioned about draining, about... It's not the chiefs who teach us, it's the co-residents who are a little older, so that's the transmission... the transmission of surgical knowledge, it's true that I forgot to tell you at the beginning, but uh... starts with the colleagues, a little older, uh... after the residency, anyway it's mutual aid (#E19)

No competitions for 3 reasons...
The first one is that we have different interests, that we don't compete on uh... on blocks or on interventions because uh... each one uh... each one has his particular interest what. The second time is that we are staggered in time, and as we are not all at the same time, uh... in the end the leaders don't necessarily have to deal with two people who have the same degree of training, so uh... no competition... And finally, the emulation, it's clear that there is some, it's clear that there is some, because when you see the... the colleague doing things uh... doing things uh... quite nice, you want to do the same so you want to... we want to know how to do, uh, as well as him or better, we want to put our hand to the wheel on the same kind of intervention and... it's a feeling that is... that is healthy what, to want to do like the other. (#E19)

Emulation
yes, there's emulation too, when you see the... the... the colleague from the year above doing a... pretty, interesting block, in autonomy, well... we'd like to do the same and we're anxious to do the same. (#E19)

With an old resident...
It's really cool because being in the operating room with one of your co-residents, I like it a lot, I think it allows you to have a bit of an outside view and to see how he does things, whereas you've learned, for example, sometimes with the same people and... and in the department, well, at the beginning, of course, you've got... you've got an older resident who explains how things work. (#E21)

Well, typically, in my first semester, something that now takes me five minutes to do took me an hour, which meant that I didn't sleep during my shifts, because in fact everything takes longer, you don't know, you don't know, you think, you ask yourself lots of questions, you. ... you have doubts, in fact, because you don't have much experience, to have an older resident telling you "no, no, but don't think forty-eight hours, uh... do this, this, this", well, you take time, and that reassures you, eh? (#E23)
